# Antagonistic Ubiquitin Switching by USP7 and RNF40 Orchestrates KDM6A Homeostasis to License Coronavirus Susceptibility

**DOI:** 10.1002/advs.202518058

**Published:** 2026-02-15

**Authors:** Meng‐Zhuo Huang, Zhong‐Yuan Yang, Shi Wang, Yan Ge, Lan Lin, Mia Madel Alfajaro, Renata B. Filler, Wan‐Yao Song, Ming‐Zhu Kong, Jin Wei

**Affiliations:** ^1^ State Key Laboratory of Virology and Biosafety Wuhan University Wuhan China; ^2^ Department of Laboratory Medicine and Immunobiology Yale School of Medicine New Haven USA

**Keywords:** Autophagy, Coronavirus, KDM6A homeostasis, Ubiquitination, Viral receptor

## Abstract

Lysine demethylase 6A (KDM6A) is a critical epigenetic regulator implicated in development, cancer, and viral infection. Although KDM6A enhances coronavirus entry by modulating viral receptor expression, the mechanisms governing its protein stability remain unknown. Here, we show that ubiquitin‐specific protease 7 (USP7) promotes diverse coronavirus infection, including SARS‐CoV, SARS‐CoV‐2, MERS‐CoV, and MHV, and represents a broad‐spectrum anti‐coronavirus target. Genetic and pharmacological inhibition of USP7 attenuates the expression of coronavirus receptors ACE2, DPP4, and Ceacam1, thereby impeding viral entry. Mechanistically, USP7 deubiquitinates KDM6A by removing K48‐linked polyubiquitin chains to prevent its proteasomal degradation. Conversely, the E3 ubiquitin ligase RNF40 catalyzes K6‐ and K11‐linked ubiquitination of KDM6A, which serves as a signal for recognition by TAX1BP1 for autophagic degradation, to restrict diverse coronavirus infection. Pharmacological inhibition of USP7 with FT671 and XL177A reduces KDM6A stability and viral receptor expression, and confers resistance to MERS‐CoV, SARS‐CoV, and all major SARS‐CoV‐2 variants of concern, including those resistant to remdesivir in primary human airway and intestinal epithelial cells. In mice, FT671 treatment was well tolerated, reduced Ceacam1 expression, and protected against MHV‐A59 infection. Collectively, our findings unveil an antagonistic ubiquitin‐mediated regulatory circuit that controls KDM6A stability, viral receptor levels, and coronavirus infection.

## Graphical abstract

We identify an antagonistic ubiquitin circuit that controls cellular permissiveness to multiple coronaviruses. The deubiquitinase USP7 stabilizes the epigenetic regulator KDM6A to upregulate viral receptors, while the E3 ligase RNF40 degrades KDM6A by catalyzing K6/K11‐ubiquitin chains of KDM6A for autophagic degradation. This switch represents a host‐directed, broad‐spectrum antiviral target.

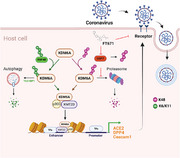

## 1 Introduction

The coronavirus disease 2019 (COVID‐19) pandemic continues to be a major threat to public health. The evolution of SARS‐CoV‐2 resulted in reduced efficacy for current authorized vaccines [1, 2], monoclonal antibodies [3, 4, 5, 6], and antiviral drugs [7, 8, 9], which highlights the need for broad‐spectrum antiviral strategies against current and emerging coronaviruses. Understanding of mechanisms of host factors in viral infection and pathogenesis is critical for the development of antiviral strategies [10, 11]. Host‐directed therapies offer particular promise as they impose higher genetic barriers to resistance and may retain efficacy against future emerging coronaviruses.

Coronavirus infection is initiated by the binding of viral spike glycoproteins to specific host receptors, a critical first step in the viral life cycle [12, 13]. Among the key receptors identified, angiotensin‐converting enzyme 2 (ACE2) serves as the primary entry receptor for SARS‐CoV, SARS‐CoV‐2, and HCoV‐NL63 [14, 15, 16]. while dipeptidyl peptidase‐4 (DPP4) mediates entry of MERS‐CoV [17]. Other coronaviruses rely on distinct receptors, aminopeptidase‐N (APN) for HCoV‐229E [18]. TMPRSS2 for HCoV‐HKU1 [19, 20], and carcinoembryonic antigen‐related cell adhesion molecule1 (Ceacam1) for mouse hepatitis virus (MHV) [21]. Growing evidence suggests that modulating the expression of these receptors may offer a viable strategy for preventing coronavirus infection. Our groups and others have shown that epigenetic regulators, including the H3K27 demethylase KMD6A (also known as UTX), SWI/SNF chromatin remodeling complex (BAF complex), and BRD2, orchestrate the expression of coronavirus receptors by mediating chromatin accessibility and enhancer activation [22, 23, 24, 25, 26]. Specifically, KDM6A forms a complex with lysine methyltransferase KMT2D and acetyltransferase p300 to promote chromatin accessibility at receptor gene loci. However, the upstream mechanisms that regulate KDM6A protein homeostasis remain poorly understood.

Protein homeostasis is essential for maintaining proper physiological functions, and ubiquitination represents one of the most important post‐translational mechanisms regulating protein stability [27]. Ubiquitination is catalyzed through a sequential enzymatic cascade involving ubiquitin‐activating (E1), ubiquitin‐conjugating (E2), and ubiquitin‐ligase (E3) enzymes, which mediate the attachment of ubiquitin to substrate proteins [28]. Ubiquitin contains seven lysine (K) residues (K6, K11, K27, K29, K33, K48, and K63), all of which can form distinct isopeptide‐linked ubiquitin chains that dictate substrate fate [29]. Deubiquitinating enzymes (DUBs) counteract this process by removing ubiquitin moieties, thereby fine‐tuning protein stability and function [27]. Among DUBs, ubiquitin‐specific protease 7 (USP7) is a widely studied cysteine protease that plays diverse roles in cellular regulation [30]. USP7 interacts with and deubiquitinates a broad array of substrates, including transcription factors, tumor suppressors, cell‐cycle regulators, epigenetic modifiers, and viral proteins [30, 31, 32, 33, 34]. Thus, USP7 is implicated in various pathological processes such as cancer, inflammatory responses, and viral infection. Nevertheless, its specific role in coronavirus infection remains unclear.

Here, we unveil an antagonistic ubiquitin‐mediated regulatory circuit that dynamically controls KDM6A stability and thereby modulates cellular permissiveness to diverse coronaviruses. We demonstrate that USP7 stabilizes KDM6A by removing K48‐linked polyubiquitin chains, consequently upregulating ACE2, DPP4, and Ceacam1 expression to promote viral entry. Conversely, the E3 ubiquitin ligase RNF40 catalyzes K6‐ and K11‐linked ubiquitination of KDM6A, triggering TAX1BP1‐dependent autophagic degradation and restricting viral infection. Pharmacological inhibition of USP7 with the small molecule inhibitor FT671 or XL177A reduces KDM6A stability and viral receptor expression, conferring protection against multiple coronaviruses, including SARS‐CoV‐2 variants resistant to current therapeutics in primary human airway and intestinal cells. In vivo, FT671 treatment is well tolerated, decreases Ceacam1 expression, and protects mice against MHV‐A59 infection. Our findings identify USP7 as a novel host‐directed target against current and future coronavirus threats through its regulation of KDM6A homeostasis.

## 2 Results

### 2.1 USP7 is Required for Highly Pathogenic Coronavirus Infection

**FIGURE 1:**
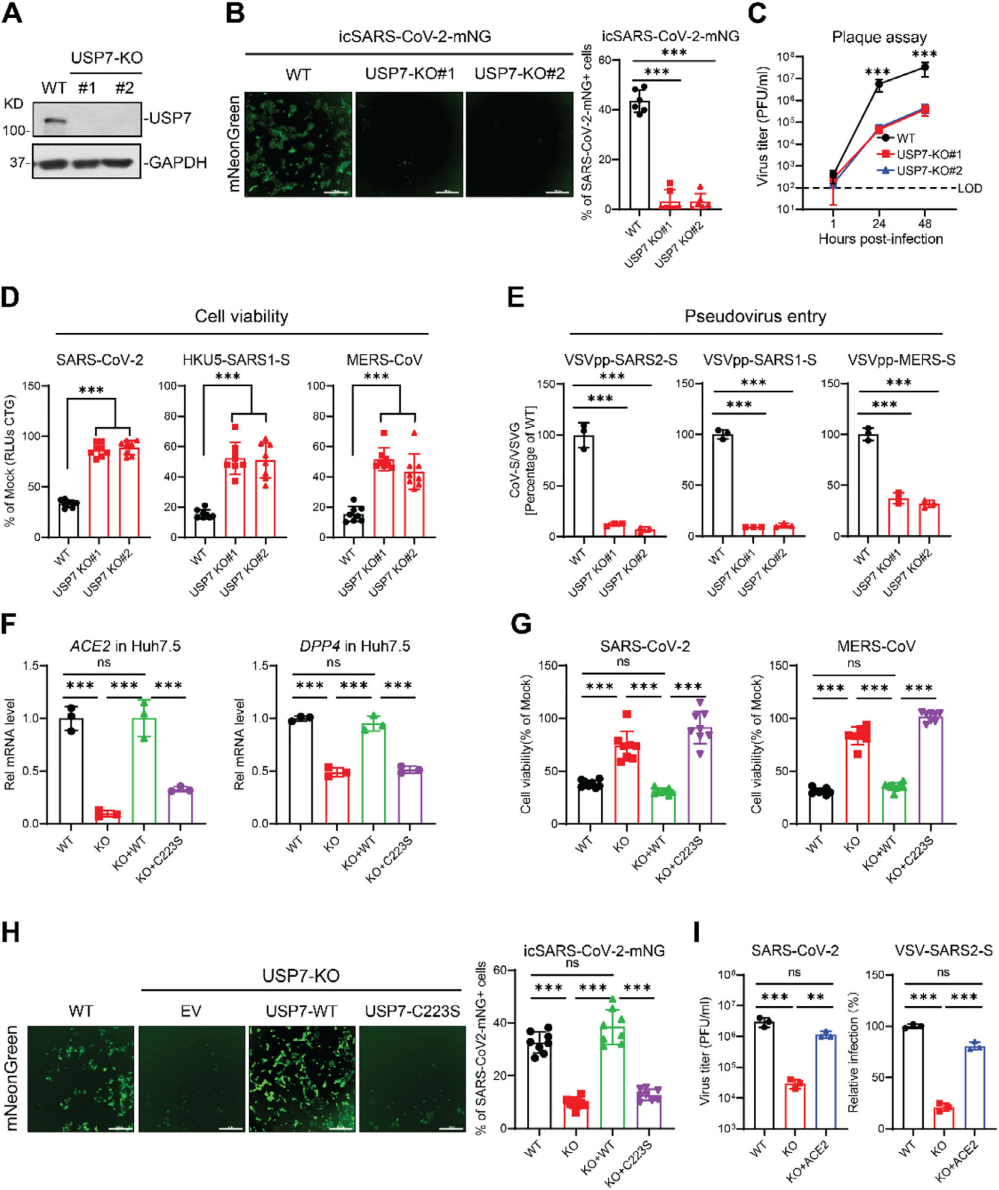
USP7 regulates ACE2 and DPP4 expressions to promote viral infection. (A) Western blot analysis for USP7 expression in Wild‐type (WT) Huh7.5 and USP7 KO clones. (B) Viral replication in WT and USP7 KO Huh7.5 cells. Cells were infected with icSARS‐CoV‐2‐mNG and then measured by fluorescence microscopy at 2 dpi. n = 6; Scale bar: 300 µm. (C) Viral production in WT and USP7 KO Huh7.5 cells. Cells were infected with SARS‐CoV‐2 at a MOI of 0.1, then the virus production was measured by plaque assays in Vero E6 cells. n = 3. Dash line, limit of detection. (D) Virus induced cell death in WT and USP7 KO cells. Cells were infected with SARS‐CoV‐2 (left), HKU5‐SARS‐CoV‐1‐S (middle), and MERS‐CoV (right) at an MOI of 0.2. Cell viability was measured by CellTiter Glo at 3 dpi. n = 8. (E) Pseudovirus entry in WT and USP7 KO cells. Cells were infected with VSV pseudotyped SARS‐CoV‐2‐S, SARS‐CoV‐1‐S, and MERS‐CoV‐S, and then the pseudovirus entry was measured by luciferase activity at 1 dpi. n = 3. (F) ACE2 and DPP4 mRNA levels were measured by qPCR in reconstituted USP7 KO cells. n = 3. (G) Cell viability in reconstituted USP7 KO Huh7.5 cells. Cells were infected with SARS‐CoV‐2 (left) and MERS‐CoV (right) at an MOI of 0.2. Cell viability was measured by CellTiter Glo at 3 dpi. (H) Viral replication in reconstituted USP7 KO cells. Cells were infected with icSARS‐CoV‐2‐mNG and measured by microscopy at 2 dpi. n = 8; Scale bar: 300 µm. (I) Overexpression of human ACE2 in USP7 KO Huh7.5 cells and infected the cells with SARS‐CoV‐2 and VSV pseudoviruses SARS‐CoV‐2‐S respectively. Viral infection was measured by plaque assays (left) and pseudovirus entry respectively. n = 3. The bar graphs show the mean ± SD of the indicated independent experiments. n, number of independent experiments; One‐way ANOVA with Tukey's test for (B to I); ^∗∗∗^ *p* < 0.001; ns, not significant.

Recently, we and others performed loss of function CRISPR screens for highly pathogenic coronaviruses in monkey and human cells and identified critical host factors for CoV infection [22, 35, 36, 37]. However, whether and how essential genes in cell proliferation such as USP7 regulate coronavirus infection remain to be determined. To investigate the role of USP7 in SARS‐CoV‐2 infection, we generated USP7 polyclonal KO cells in Vero E6 cells using CRISPR‐Cas9 (Figure S1A). We challenged USP7 polyclonal KO cells with icSARS‐CoV‐2‐mNG virus expressing a mNeonGreen reporter and quantified the frequency of infected cells by microscopy. Disruption of USP7 inhibited icSARS‐CoV‐2‐mNG infection compared to control cells (Figure S1B). To investigate the role of USP7 in human cells, we generated two independent USP7 KO clones in Huh7.5 cells and confirmed knockout efficiency by western blot (Figure 1A). Again, inactivation of USP7 reduced SARS‐CoV‐2 replication and virus production compared to wild‐type (WT) cells as determined by microscopy and plaque assay, respectively (Figure 1B,C).

Next, we investigate the role of USP7 on other coronavirus infection. We examined diverse coronavirus replication in USP7 KO cells, including a bat coronavirus HKU5 expressing SARS‐CoV‐1 spike (HKU5‐SARS1‐S), SARS‐CoV‐2, or MERS‐CoV. Inactivation of USP7 reduced HKU5‐SARS1‐S, SARS‐CoV‐2, and MERS‐CoV induced cell death as measured by cell viability and viral production (Figure 1D; Figure S1C). Next, we investigated whether USP7 regulates viral entry by coronavirus spike pseudotyped viruses. Inactivation of USP7 blocked SARS‐CoV‐1, SARS‐CoV‐2 and MERS‐CoV spike pseudotyped virus entry (Figure 1E). Taken together, these results demonstrate that USP7 plays critical role in highly pathogenic coronavirus infection.

### 2.2 Deubiquitinase Activity of USP7 Is Required for Regulation of ACE2 and DPP4 Expression

To investigate how USP7 regulates viral entry, we first analyzed the expression of coronavirus receptors. Genetic inactivation of USP7 reduced ACE2 and DPP4 mRNA levels, as measured by qPCR, and decreased ACE2 protein expression, as assessed by western blot (Figure S1D,E). However, DPP4 protein was undetectable in Huh7.5 cells, falling below the detection limit, therefore, its expression could not be evaluated in USP7 knockout cells.

To determine whether the deubiquitinase activity of USP7 is required for its proviral activity, we reintroduced the full length USP7 (WT), the deubiquitinase inactive USP7 mutant (C223S) or empty vector (EV) in USP7 KO clone and the expression levels were confirmed by western blot (Figure S1F). We first examined the viral receptor expression in rescued cell lines. Reintroduction of WT USP7, but not the C223S mutant restored ACE2 and DPP4 expression in USP7 KO cells (Figure 1F). Consistently, reintroduction of USP7 WT, but not C223S mutant restored the SARS‐CoV‐2 replication, virus‐induced cell death and pseudovirus entry (Figure 1G,H; Figure S1G). Taken together, these data demonstrate that USP7 promotes highly pathogenic coronavirus infection in a deubiquitinase activity‐dependent manner.

Given that viral receptor expressions are downregulated following USP7 deletion, we sought to determine if viral entry could be rescued in USP7 KO cells following exogenous expressions of human ACE2 and DPP4. Lentiviral expressions of human ACE2 and DPP4 in USP7 KO cells restored viral infection, as well as pseudovirus entry respectively (Figure 1I; Figure S1H,I), underscoring that USP7‐mediated expression of ACE2 and DPP4 are responsible for the proviral phenotype observed.

### 2.3 USP7 Deubiquitinates and Stabilizes KDM6A

**FIGURE 2:**
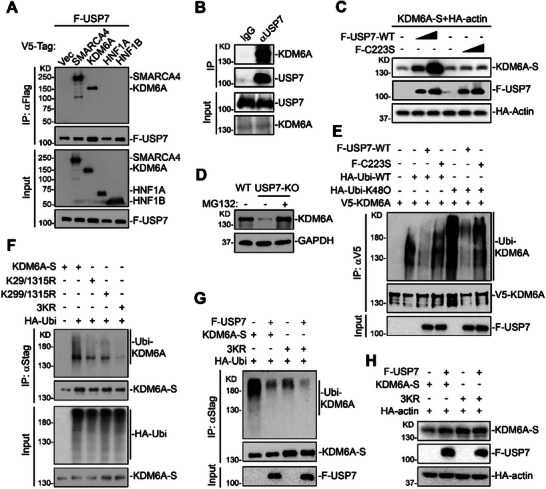
USP7 deubiquitinates and stabilizes KDM6A. (A) USP7 interacts with KDM6A. 293T cells were co‐transfected with plasmids encoding Flag‐USP7 and V5‐tagged nuclear proteins. At 24 h post‐transfection, cells were processed for coimmunoprecipitation and immunoblot analysis. (B) Endogenous association of USP7 with KDM6A. Huh7.5 cells were processed for Co‐IP and immunoblot analysis. (C) USP7 enhances the KDM6A protein level in a dose‐dependent manner. 293T cells were transfected with KDM6A‐S and HA‐actin together with increasing doses of Flag‐USP7 and Flag‐USP7‐C223S for 24 h before immunoblot analysis. HA‐actin was used as the transfection and loading control. (D) MG‐132 treatment rescues KDM6A expression in USP7 KO cells. USP7 KO cells were treated with DMSO or MG132 for 6 h before immunoblot analysis. (E) USP7 catalyzes the removal of K48‐linked polyubiquitin chains of KDM6A. 293T cells were transfected with the indicated plasmids for 24 h before immunoprecipitation and immunoblot analysis. (F) USP7 catalyzes the removal of polyubiquitin chains of KDM6A at Lys29, Lys299, and Lys1315 residues. 293T cells were transfected with WT KDM6A and KR mutants together with HA‐ubiquitin for 24 h before coimmunoprecipitation and immunoblot analysis. (G) The effects of USP7 on ubiquitination of KDM6A WT and 3KR mutant. 293T cells were transfected with the indicated plasmids for 24 h before coimmunoprecipitation and immunoblot analysis. (H) Effects of USP7 on stability of KDM6A WT and 3KR mutant. 293T cells were transfected with the indicated plasmids for 24 h before immunoblot analysis. HA‐actin was used as the transfection and loading control.

We next asked what the target is for USP7. Recently, we and others have identified numerous transcription factors (TFs) and epigenetic regulators for ACE2 and DPP4, including DYRK1A, HNF1, BAF complex, and KDM6A complex [22, 23, 24, 38, 39]. Specifically, KDM6A regulates ACE2 and DPP4 enhancer activation by recruiting KMT2D and p300 independent of complex with DYRK1A and/or BAF complex. USP7 is critical for multiple nuclear protein homeostasis [31, 32]. We hypothesized that USP7 may promote viral receptor expression and viral infection by mediating the critical stability of other host factors. We next determined whether USP7 was able to bind these known TFs or epigenetic regulators. Co‐IP experiments indicated that USP7 interacted with KDM6A and SMARCA4, but not the TFs HNF1A and HNF1B (Figure 2A). Consistent with this data, immunoprecipitation of endogenous KDM6A captured USP7 in Huh7.5 cells (Figure 2B). Given that inactivation of USP7 phenocopied KDM6A deficiency but not SMARCA4 on diverse coronavirus infection, we hypothesis that USP7 targets KDM6A for viral infection. We next determined KDM6A expression in presence or absence of USP7. Overexpression of USP7 WT, but not C223S mutant stabilized KDM6A in a dose‐dependent manner (Figure 2C). In contrast, the genetic inactivation of USP7 reduced KDM6A protein level, whereas the mRNA level of KDM6A was unaffected upon USP7 deletion (Figure 2D; Figure S1D). Proteasome inhibitor MG‐132 treatment restored KDM6A expression in USP7 KO cells (Figure 2D), suggesting that USP7 may regulate ubiquitin‐mediated proteasomal degradation of KDM6A.

Given that USP7 interacts with KDM6A, and its deubiquitinating enzymatic activity is required for receptor expression and viral entry, we tested whether USP7 eliminates polyubiquitin chains from KDM6A. As expected, we found that WT USP7 but not the enzymatic inactive mutant (C223S) catalyzed the removal of K48‐linked polyubiquitin chains from KDM6A (Figure 2E). Mutation of Lys29, Lys299, and Lys1315 to Arg of KDM6A (3KR) abolished polyubiquitination of KDM6A (Figure 2F). In addition, the 3KR mutant of KDM6A is insensitive to USP7‐mediated deubiquitination and stability (Figure 2G,H).

**FIGURE 3:**
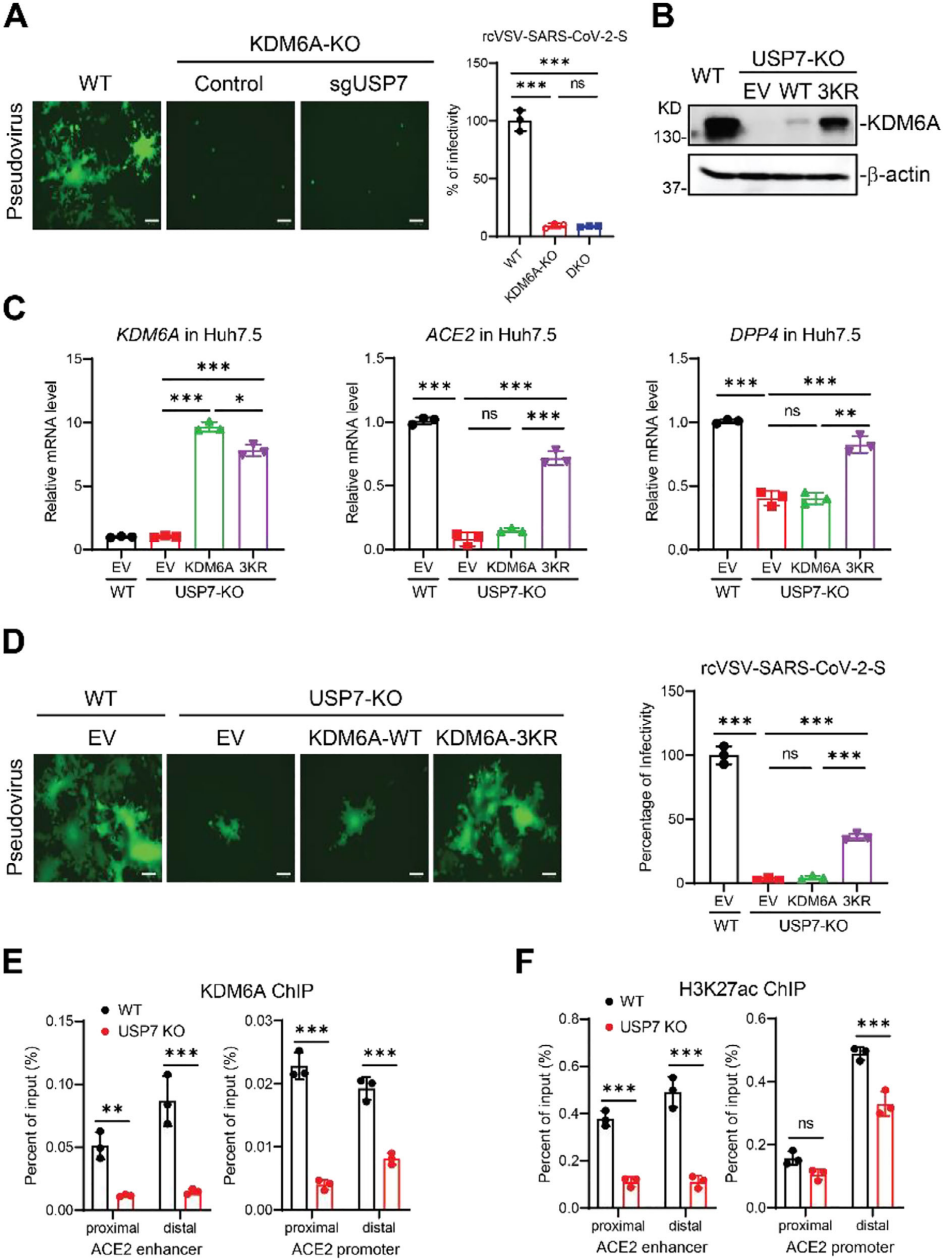
USP7 mediated KDM6A stability is required for viral receptor expression and viral infection. (A) rcSARS‐CoV‐2‐S replication in KDM6A or USP7/KDM6A double KO cells. KDM6A KO clone was transduced with lentivirus containing USP7‐sgRNA, then infected with rcVSV‐SARS‐CoV‐2‐S. Infected cells were imaged via fluorescence microscopy (left), and GFP expressing cell frequency was measured 1 dpi (right). n = 3. Scale bar: 100 µm. (B) KDM6A expression in USP7 KO cells by lentiviral transduction of empty vector (EV), KDM6A WT and 3KR mutant. Cells were transduced with empty vector, KDM6A WT or 3KR mutant by lentiviral‐mediated gene transfer. Cells were selected with antibiotics for 5 days before immunoblot analysis. (C) Effects of KDM6A on ACE and DPP4 expression in USP7 KO cells. USP7 KO cells were transduced with EV, KDM6A WT or 3KR mutant by lentiviral‐mediated gene transfer. The cells were processed for qPCR experiments. n = 3. (D) Effects of KDM6A and 3KR mutants on SARS‐CoV‐2 pseudovirus infection. Cells were infected with rcVSV‐SARS‐CoV‐2 and measured by microscopy at 2 dpi. n = 3. Scale bar: 100 µm. (E‐F) ChIP‐qPCR assays were performed using KDM6A (E) and H3K27ac (F) antibody in WT and USP7 KO Huh7.5 cells to detect the binding sites in ACE2 proximal and distal enhancer regions and promoter regions. The bar graphs show the mean ± SD of three independent experiments. n, number of independent experiments; Unpaired Student's t test for (E and F); One‐way ANOVA with Tukey's test for (A, C, and D); ^∗^ *p* < 0.05; ^∗∗^ *p* < 0.01; ^∗∗∗^ *p* < 0.001; ns, not significant.

To confirm that USP7 regulates viral infection through KDM6A, we depleted USP7 in KDM6A KO clones. Notably, we observed similar reduction of viral replication in KDM6A single KO and USP7/KDM6A double KO cells (Figure 3A), suggesting USP7‐mediated KDM6A stability specifically is responsible for the proviral activity observed. To determine if viral infectivity could be rescued in USP7 KO cells after rescue with KDM6A 3KR mutant, we introduced WT KDM6A, 3KR mutant or empty vector into USP7 KO cells by lentiviral transduction and confirmed expression by western blot (Figure 3B). The KDM6A protein levels were much lower in the introduction of KDM6A WT compared to 3KR mutant, despite similar KDM6A mRNA levels (Figure 3B,C). Notably, the marked increase in protein levels of the ubiquitination‐resistant KDM6A mutant relative to WT, in the absence of a corresponding increase in mRNA, further underscores the potent destabilizing effect exerted by USP7 on wild‐type KDM6A. Importantly, the introduction of KDM6A 3KR mutant, but not WT KDM6A, rescued ACE2 and DPP4 expression in USP7 KO cells (Figure 3C). Consistently, lentiviral expression of KDM6A 3KR mutant, but not WT KDM6A, restored rcVSV‐SARS‐CoV‐2‐S infection (Figure 3D).

Given that KDM6A is critical for setting up the active state of enhancers, we next determined the occupancy of KDM6A at the enhancer and promoter loci of *ACE2* gene. To do this, we performed chromatin‐immunoprecipitation (ChIP) of KDM6A and active enhancer marker H3K27ac in WT and USP7 KO cells. Genetic inactivation of USP7 reduced the occupancy of KDM6A at both the promoter and enhancer regions of *ACE2* (Figure 3E). Concomitantly, the levels of the active enhancer mark H3K27ac at these loci were also diminished in USP7 KO cells compared to WT cells (Figure 3F). Taken together, USP7 promotes highly pathogenic coronavirus infection by deubiquitinating and stabilizing KDM6A, which in turn is required for its proper recruitment and function at viral receptor gene loci.

### 2.4 RNF40 Ubiquitinates and Degrades KDM6A to Restrict Viral Infection

**FIGURE 4:**
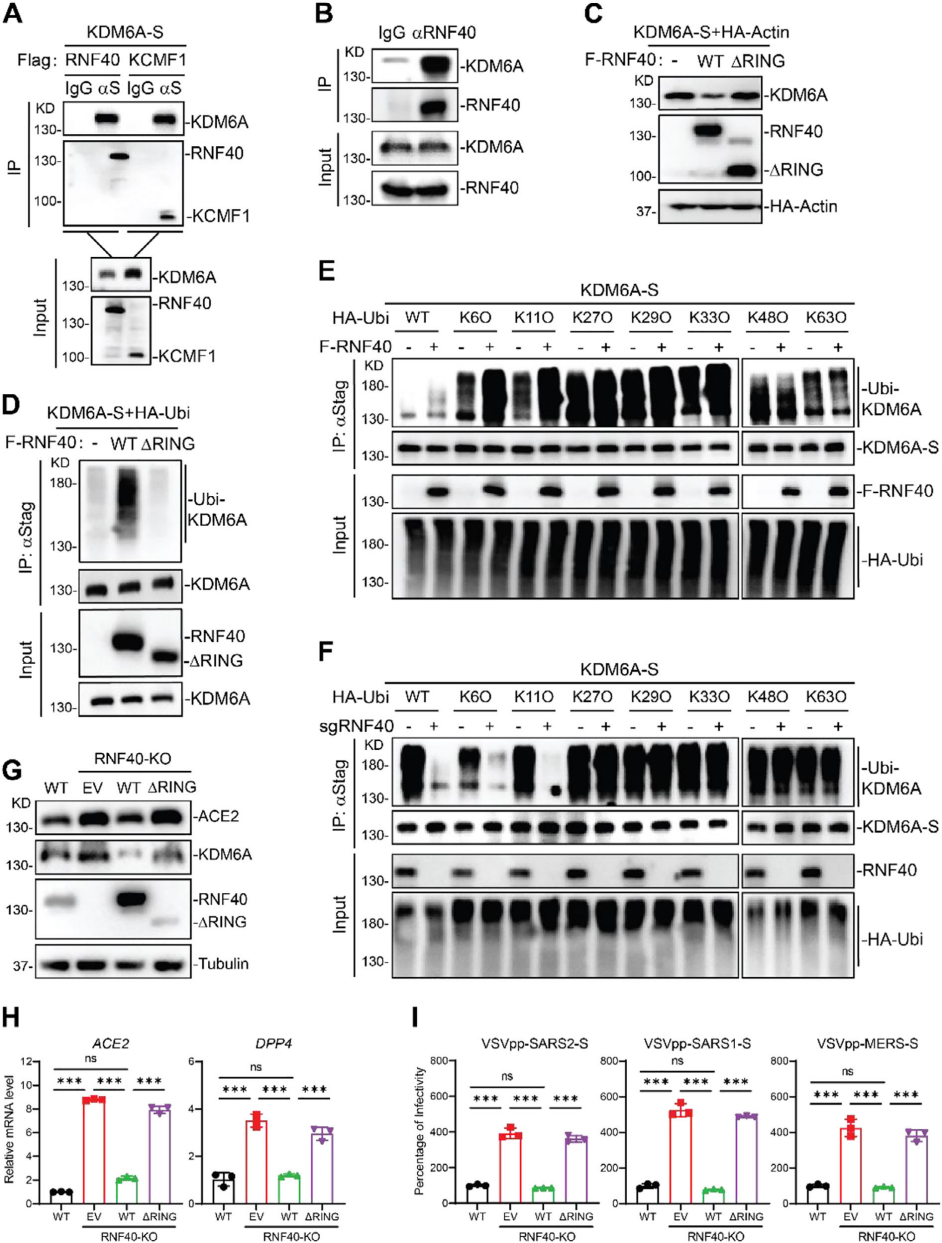
RNF40 ubiquitinates and degrades KDM6A to restrict viral infection. (A) RNF40 interacts with KDM6A. 293T cells were transfected with KDM6A‐S together with Flag‐RNF40 or Flag‐KCMF1 for 24 h before coimmunoprecipitation and immunoblot assays. (B) Endogenous association of RNF40 with KDM6A. Huh7.5 cells were collected for co‐immunoprecipitation and immunoblot assays. (C) Effects of overexpression of RNF40 or ΔRING mutant on KDM6A expression. 293T cells were transfected with KDM6A‐S and HA‐actin together with empty vector, Flag‐RNF40 or ΔRING mutant for 24 h before immunoblot analysis. HA‐actin was used as the transfection and loading control. (D) RNF40 catalyzes polyubiquitination of KDM6A. 293T cells were transfected with HA‐ubiquitin and KDM6A‐S together with empty vector, F‐RNF40 or ΔRING mutant for 24 h before ubiquitination assays. (E) RNF40 catalyzes K6‐ and K11‐linked ubiquitination of KDM6A. 293T cells were transfected with the indicated plasmids for 24 h before ubiquitination assays. (F) RNF40 deficiency impairs the K6‐ and K11‐linked ubiquitination of KDM6A. Control or RNF40 KO cells were transfected with the indicated plasmids for 24 h before ubiquitination assays. (G) Effects of RNF40 and ΔRING mutant on KDM6A protein expression in RNF40 KO Huh7.5 cells. RNF40 KO cells were reconstituted with empty vector, WT RNF40 or ΔRING, then cells were selected with blasticitin for 5 days before immunoblot analysis. (H) ACE2 and DPP4 mRNA expression in reconstituted RNF40 KO Huh7.5 cells. RNF40 KO cells reconstituted with empty vector, RNF40 or ΔRING as in (G) were processed for qPCR experiments. n = 3. (I) Pseudovirus entry in reconstituted RNF40 KO Huh7.5 cells. Cells were infected with VSV‐GFP‐pseudotyped SARS‐CoV‐2‐S, SARS‐CoV‐S or MERS‐CoV‐S for 24 h before microscopy analysis. n = 3. The bar graphs show the mean ± SD of three independent experiments. n, number of independent experiments; One‐way ANOVA with Tukey's test for (H and I); ^∗∗∗^ *p* < 0.001; ns, not significant.

We next sought to identify the E3 ligase(s) that mediates KDM6A stability. To do this, we analyzed the KDM6A interactomes dataset in human prostate cancer cells and identified three E3 ligases RNF40, HUWE1, and KCMF1, which interacted with KDM6A (Figure S2A) [40]. We performed immunoprecipitation assays and confirmed that KDM6A was associated with RNF40 and KCMF1 (Figure 4A). Immunoprecipitation of endogenous RNF40 captured KDM6A in Huh7.5 cells (Figure 4B). Moreover, overexpression of RNF40 but not KCMF1 reduced the KDM6A stability in a dose‐dependent manner (Figure S2B). Similarly, overexpression of RNF40 reduced endogenous KDM6A protein levels (Figure S2C). Previous studies have shown that RNF40 complexes with RNF20 and UbcH6 to regulate the H2B monoubiquitination [41, 42]. We next determined whether the ability of RNF40 to degrade KDM6A requires its association with RNF20. Unexpectedly, overexpression of RNF20 has no effect on KDM6A stability (Figure S2D), suggesting that RNF40 regulates KDM6A stability independent of RNF20.

Because RNF40 is an E3 ubiquitin ligase, we determined whether the E3 ligase activity is required for KDM6A degradation. Deletion of the RING domain, which is critical for its E3 ligase activity, impaired its ability to associate and degrade KDM6A (Figure 4C; Figure S2E). We next examined whether RNF40 catalyzes polyubiquitination of KDM6A. As expected, we found that WT RNF40, but not the enzyme‐inactive mutant (ΔRING), promoted polyubiquitination of KDM6A (Figure 4D). We next examined the ubiquitination types of KDM6A catalyzed by RNF40. The ubiquitination assays indicated that RNF40 catalyzed K6‐ and K11‐linked polyubiquitination of KDM6A (Figure 4E). On the contrary, disruption of RNF40 abolished K6‐ and K11‐linked polyubiquitination of KDM6A (Figure 4F). These data demonstrate that RNF40 targets KDM6A for ubiquitination and degradation.

To define the potential role of RNF40 in maintaining KDM6A stability and viral infection, we generated RNF40 polyclonal KO cells and confirmed the knockout efficiency by western blot (Figure S3A). Disruption of RNF40, but not KCMF1, significantly promoted KDM6A expression at protein level, whereas the KDM6A mRNA level was unaffected (Figure S3A,B). Given the role of KDM6A in mediating ACE2 and DPP4 expression, we evaluated the impact of RNF40 disruption on ACE2 and DPP4 mRNA levels in Huh7.5 cells. ACE2 and DPP4 mRNA levels were upregulated in RNF40‐disrupted cells but not in KCMF1‐disrupted cells (Figure S3B). Notably, disruption of RNF40 promoted SARS‐CoV‐1, SARS‐CoV‐2, and MERS‐CoV entry as measured by pseudovirus entry assay (Figure S3C). To test whether RNF40‐mediated degradation of KDM6A is responsible for the antiviral phenotype, we reintroduced wild‐type RNF40, the enzyme‐inactive mutant (ΔRING) or empty vector into RNF40 KO cells and confirmed expression by western blot (Figure 4G). Reintroduction of WT RNF40, but not ΔRING mutant, rescued KDM6A and ACE2 protein expression (Figure 4G). We next examined the viral receptor expression at the mRNA level in rescued cell lines. Reintroduction of WT RNF40, but not ΔRING mutant, restored ACE2 and DPP4 expression in RNF40 KO cells (Figure 4H). Consistently, reintroduction of WT RNF40, but not ΔRING mutant, restored the SARS‐CoV‐1, SARS‐CoV‐2 and MERS‐CoV infection (Figure 4I; Figure S3D). Collectively, RNF40 targets KDM6A for ubiquitination and degradation to reduce viral receptor expression and viral infection.

### 2.5 RNF40 Degrades KDM6A Through TAX1BP1‐Mediated Autophagy

**FIGURE 5:**
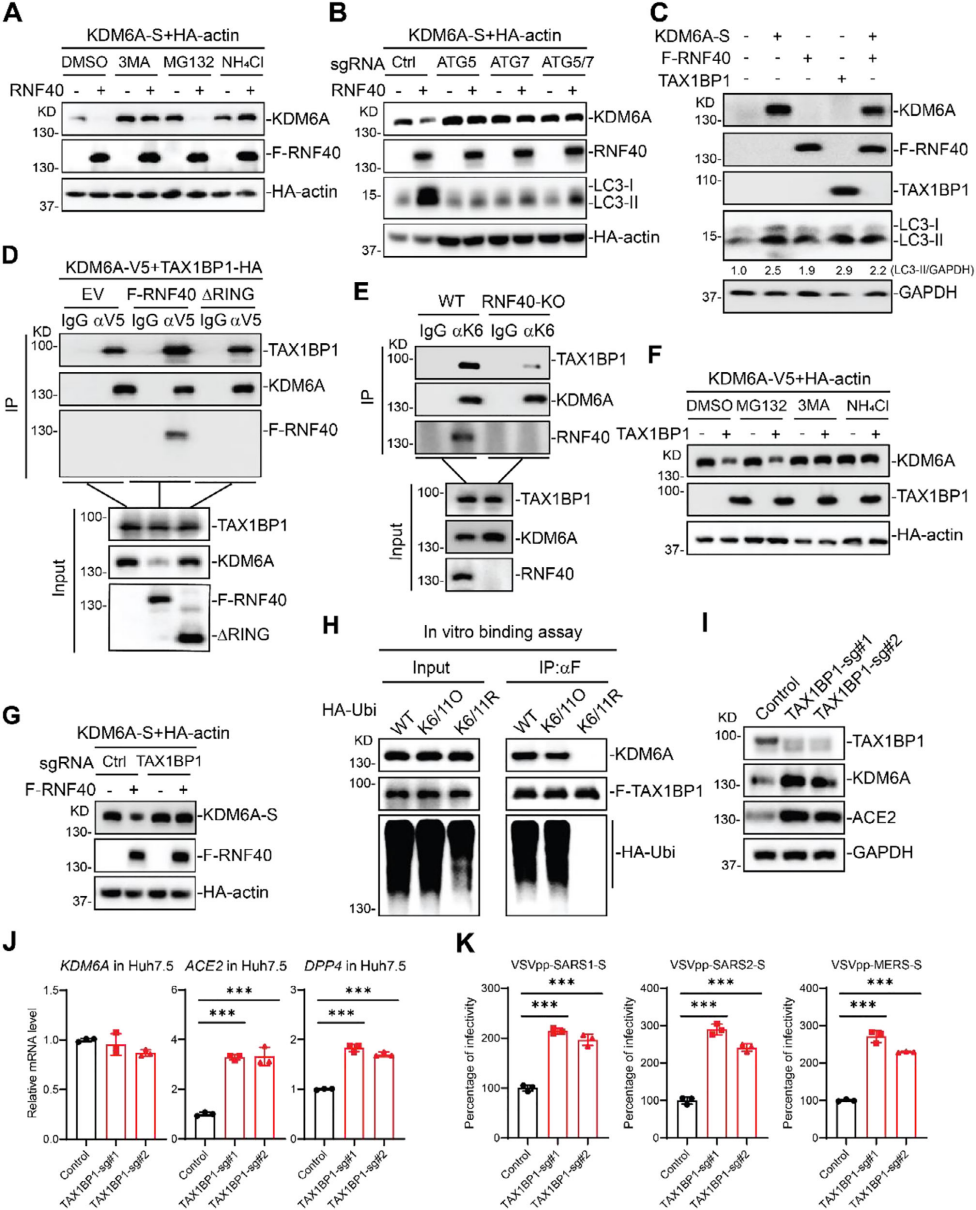
RNF40 degrades KDM6A through TAX1BP1‐mediated autophagy. (A) Effects of MG‐132, 3MA, and NH_4_Cl on KDM6A degradation induced by RNF40.293T cells were transfected with the indicated plasmids for 24 h and then treated with DMSO, MG‐132, 3MA or NH4Cl for 6 h before immunoblot analysis. (B) Effects of ATG5 or ATG7 deficiency on KDM6A degradation induced by RNF40. Control and ATG5/7 deficient cells were transfected with KDM6A‐S and HA‐actin together with empty vector or Flag‐RNF40 for 24 h before immunoblot analysis. (C) Overexpression of RNF40 and KDM6A induces autophagy. 293T cells were transfected with Flag‐RNF40, KDM6A‐S or TAX1BP1‐HA for 24 h before immunoblot analysis. LC3‐II level is normalized to GAPDH. (D) RNF40 promotes KDM6A‐TAX1BP1 association. 293T cells were transfected with KDM6A‐V5 and TAX1BP1‐HA together with empty vector or Flag‐RNF40 and its mutant for 24 h before coimmunoprecipitation and immunoblot assays. (E) RNF40 deficiency impairs the KDM6A‐TAX1BP1 association. WT and RNF40 KO cells were collected for endogenous coimmunoprecipitation and immunoblot analysis. (F) Effects of MG‐132, 3MA, and NH_4_Cl on KDM6A degradation induced by TAX1BP1. 293T cells were transfected with the indicated plasmids for 24 h and then treated with DMSO, MG‐132, 3MA, or NH4Cl for 6 h before immunoblot analysis. (G) Effects of TAX1BP1 deficiency on KDM6A degradation induced by RNF40. Control and TAX1BP1‐deficient cells were transfected with KDM6A‐S and HA‐actin together with empty vector or Flag‐RNF40 for 24 h before immunoblot analysis. (H) TAX1BP1 recognized K6/K11‐ubiquitin conjugated KDM6A in vitro binding assays. KDM6A in vitro ubiquitination was catalyzed by using WT, K6/K11R, and K6/K11O ubiquitin plasmids. Then the ubiquitinated KDM6A was incubated with TAX1BP1 for immunoprecipitation. (I) TAX1BP1 deficiency elevated KDM6A protein level. Huh7.5 cells were transduced with TAX1BP1 sgRNAs by lentiviral‐mediated CRISPR‐Cas9 gene KO. Cells were selected with puromycin for 5 days before immunoblot analysis. (J) ACE2, DPP4 and KDM6A mRNA expression in TAX1BP1 polyclonal KO Huh7.5. CRISPR‐mediated TAX1BP1 polyclonal KO cells were processed for qPCR analysis. n = 3.(K) Pseudovirus infection in TAX1BP1 polyclonal KO cells. Cells were infected with VSVpp‐GFP pseudotyped SARS‐CoV‐S, SARS‐CoV‐2‐S, and MERS‐CoV‐S. Infected cells were imaged via fluorescence microscopy, and GFP expressing cell frequency was measured 1 dpi. n = 3.HA‐actin was used as the transfection and loading control in (A‐B) and (G‐H). The bar graphs show the mean ± SD of three independent experiments. n, number of independent experiments; One‐way ANOVA with Tukey's test for (J and K); ^∗∗∗^ *p* < 0.001.

We next sought to determine the mechanism by which the KDM6A stability is regulated by RNF40. We first examined the degradation pathway of KDM6A induced by RNF40. The autophagy inhibitor 3‐methyladenine (3MA) and lysosome inhibitor NH_4_Cl treatment restored RNF40‐mediated KDM6A degradation, whereas RNF40‐mediated KDM6A downregulation was unaffected upon proteasome inhibitor MG132 treatment (Figure 5A). To clarify the role of autophagy in KDM6A degradation induced by RNF40, we examined RNF40‐mediated KDM6A expression in ATG5 and/or ATG7‐deficient cells, which are key autophagy proteins [43, 44]. As expected, RNF40‐mediated decrease of KDM6A protein level was restored following disruption of ATG5 and/or ATG7 (Figure 5B), suggesting that RNF40 promoted autophagy‐lysosome dependent degradation of KDM6A.

We next sought to investigate the mechanism of KDM6A degradation induced by RNF40‐mediated autophagy. We first determined whether RNF40 or KDM6A could affect autophagic flux. As expectedly, overexpression of RNF40 or KDM6A alone or in combination increased LC3‐I to LC3‐II conversion (Figure 5C). Selective autophagy is initiated by recognizing cargos via autophagy receptors, including Tax1‐binding protein 1 (TAX1BP1), SQSTM1/p62, NIX, NDP52, OPTN, and TOLLIP [45, 46, 47]. All autophagy receptors can recognize ubiquitin marks attached to the cargos [48]. Given the role of RNF40 in mediating KDM6A ubiquitination, we hypothesized that RNF40 may promote degradation of KDM6A through autophagy receptors. We first determined whether KDM6A was able to bind these known autophagy receptors. Co‐IP experiments indicated that KDM6A was associated with TAX1BP1, but not the other autophagy receptors, including TOLLIP, NIX, NDP52 OPTN or p62 (Figure S4A). Moreover, we observed that RNF40 was strongly associated with TAX1BP1 and p62, but weakly associated with NDP52 (Figure S4B). To further clarify the relationship between TAX1BP1 and KDM6A degradation induced by RNF40, we first determined the KDM6A‐TAX1BP1 complex association in the presence or absence of RNF40. Overexpression of RNF40 but not ΔRING mutant promoted the association of KDM6A with TAX1BP1 (Figure 5D; Figure S4C). In contrast, RNF40 deficiency impaired the association of KDM6A with TAX1BP1 (Figure 5E; Figure S4D). Importantly, overexpression of TAX1BP1 reduced KDM6A protein level (Figure 5F). Similarly, 3MA and NH_4_Cl, but not MG132 treatment, restored TAX1BP1‐mediated KDM6A degradation (Figure 5F).

Next, we sought to investigate the relationship between K6/K11‐linked ubiquitination of KDM6A and TAX1BP1‐dependent autophagy. We first evaluated the RNF40‐mediated KDM6A degradation in TAX1BP1 disrupted cells. RNF40‐mediated decrease of KDM6A protein level was restored following disruption of TAX1BP1 (Figure 5G). To determine whether K6/K11‐conjugated KDM6A is directly recognized by TAX1BP1, we constructed the ubiquitin mutants, K6/K11 only (K6/K11O), where only Lys 6 and 11 are available for ubiquitin chain formation, and K6/K11‐deficient (K6/K11R) mutant, where Lys 6 and 11 are mutated to arginine for in vitro binding assays. As expectedly, TAX1BP1 specifically recognized and associated with KDM6A conjugated with K6/K11‐linked ubiquitin chains (Figure 5H). Collectively, RNF40 catalyzed K6/K11‐linked ubiquitin chains of KDM6A serves as a specific signal for recognition by TAX1BP1.

Finally, we determined the potential role of TAX1BP1 in maintaining KDM6A expression and viral infection. We generated two independent TAX1BP1 polyclonal KO Huh7.5 cells by CRISPR‐Cas9 technology, and the KO efficiency was confirmed by western blot (Figure 5I). As expected, disruption of TAX1BP1 elevated KDM6A protein expression as well as ACE2 expression (Figure 5I). Furthermore, disruption of TAX1BP1 upregulated ACE2 and DPP4 mRNA expression, while the KDM6A mRNA expression was unaffected as measured by qPCR (Figure 5J). Consistently, disruption of TAX1BP1 promoted SARS‐CoV, SARS‐CoV‐2, and MERS‐CoV entry and viral replication as measured by pseudovirus entry assay and qPCR, respectively (Figure 5K; Figure S4E). Taken together, RNF40 promotes KDM6A degradation via TAX1BP1‐dependent autophagy to restrict viral infection.

### 2.6 Inhibition of USP7 Abrogates ACE2, DPP4, and Ceacam1 Expression and Blocks Viral Infection

**FIGURE 6:**
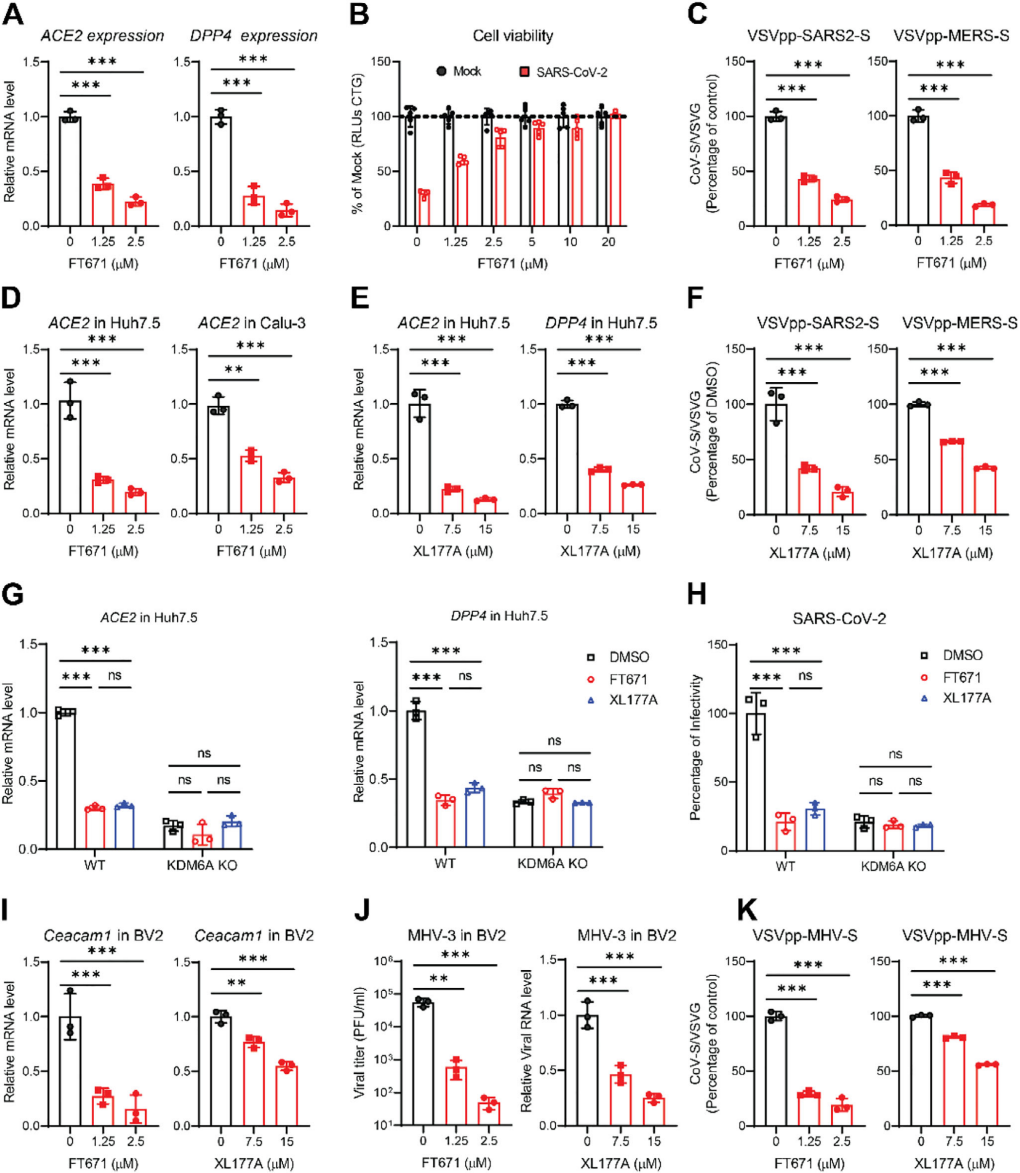
Inhibition of USP7 reduces multiple viral receptor expressions and blocks viral infection. (A) Inhibition of USP7 inhibits ACE2 and DPP4 expression. Vero E6 cells were treated with FT671 at 1.25 µM for the indicated times, and ACE2 (left) and DPP4 (right) levels were measured by qPCR. n = 3. (B) FT671 dose‐dependently inhibits SARS‐CoV‐2 induced cell death. Vero E6 were pretreated with FT671 for 48 h, then cells were infected with SARS‐CoV‐2 at an MOI of 0.2 for 3 dpi before cell viability analysis. n = 5. (C) FT671 dose‐dependently inhibits pseudovirus infection. Vero E6 cells were pretreated with 1.25 and 2.5 µM FT671 for 2 days and then infected with SARS‐CoV‐2‐S and MERS‐CoV‐S pseudoviruses. Luciferase relative to VSVpp‐VSV‐G control was measured 1 dpi. n = 3. (D) FT671 inhibits ACE2 mRNA expression in Huh7.5 and Calu‐3 cells. Huh7.5 and Calu‐3 cells were treated with DMSO or FT671 for 2 days before qPCR analysis. n = 3. (E) XL177A inhibits ACE2 and DPP4 mRNA expression in Huh7.5 cells. Huh7.5 cells were treated with DMSO or XL177A for 2 days before qPCR analysis. n = 3. (F) XL177A dose‐dependently inhibits pseudovirus entry. Huh7.5 cells were pretreated with 7.5 and 15 µM XL177A for 2 days and then infected with SARS‐CoV‐2‐S and MERS‐CoV‐S pseudoviruses. Luciferase relative to VSVpp‐VSV‐G control was measured 1 dpi. n = 3. (G) KDM6A deficiency abolishes the inhibition of virial receptor expression following USP7 inhibitor treatment. WT and KDM6A KO cells were treated with FT671 and XL177A before qPCR analysis was performed. (H) KDM6A deficiency abolishes the inhibition of viral infection following USP7 inhibitor treatment. WT and KDM6A KO cells were treated with FT671 and XL177A, then infected with SARS‐CoV‐2 for 24 h before viral infection was measured. (I) FT671 and XL177A inhibit Ceacam1 expression in BV2 cells. BV2 cells were treated with FT671 or XL177A for 2 days before qPCR analysis. n = 3. (J) FT671 and XL177A inhibit MHV‐3 infection in BV2 cells. BV2 cells were pretreated with FT671 or XL177A, then infected with MHV‐3 for 24 hpi. Virus production and viral replication were measured by plaque assay and qPCR, respectively. n = 3. (K) FT671 inhibits MHV pseudovirus infection in BV2 cells. BV2 cells were pretreated with FT671 for 48 h and then infected with MHV‐A59‐S pseudovirus. Luciferase relative to VSVpp‐VSV‐G control was measured 1 dpi. n = 3. The bar graphs show the mean ± SD of three independent experiments. n, number of independent experiments; One‐way ANOVA with Tukey's test for (A to K); ^∗∗^ *p* < 0.01; ^∗∗∗^ *p* < 0.001; ns, not significant.

To investigate the potential of USP7 as a host‐directed therapeutic target for human coronaviruses, we determined the effect of a potent and selective catalytic inhibitor of USP7, FT671, on SARS‐CoV‐2 infection. We first examined the impact of FT671 on viral receptor expression. Notably, inhibition of USP7 by FT671 inhibited ACE2 and DPP4 expression at mRNA and protein levels in African green monkey Vero E6 cells (Figure 6A; Figure S5A,B). Consistent with this, FT671 inhibited SARS‐CoV‐2 prototypic strain‐induced cell death and viral entry in a dose‐dependent manner (Figure 6B,C). Moreover, we observed similar inhibition of ACE2 expression in Huh7.5 and Calu‐3 cells. (Figure 6D). To determine the USP7 inhibitor specificity, we used another selective and structurally distinct irreversible USP7 inhibitor XL177A. Consistently, XL177A treatment inhibited ACE2 and DPP4 expression in a dose‐ and time‐dependent manner (Figure 6E; Figure S5C,D), as well as the pseudovirus entry (Figure 6F). To genetically link the inhibitors’ effect to KDM6A, we treated KDM6A KO cells with FT671 and XL177A. Genetic inactivation of KDM6A completely abolished the ability of FT671 and XL177A to downregulate viral receptor expression and viral infection (Figure 6G,H). Collectively, these findings suggest that the antiviral phenotype of USP7 inhibitors is specifically due to on‐target inhibition of USP7 and its downstream regulation of KDM6A.

Given that USP7 maintains KDM6A, which is critical for mouse hepatitis virus (MHV) infection [38], we investigated whether inhibition of USP7 reduces MHV receptor expression and viral infection. As expected, inhibition of USP7 enzymatic activity by FT671 or XL177A dose‐dependently inhibited Ceacam1 expression and viral infection (Figure 6I–K).

### 2.7 USP7 Inhibition Blocks SARS‐CoV‐2 and MERS‐CoV Infection in Primary Human Cells

**FIGURE 7:**
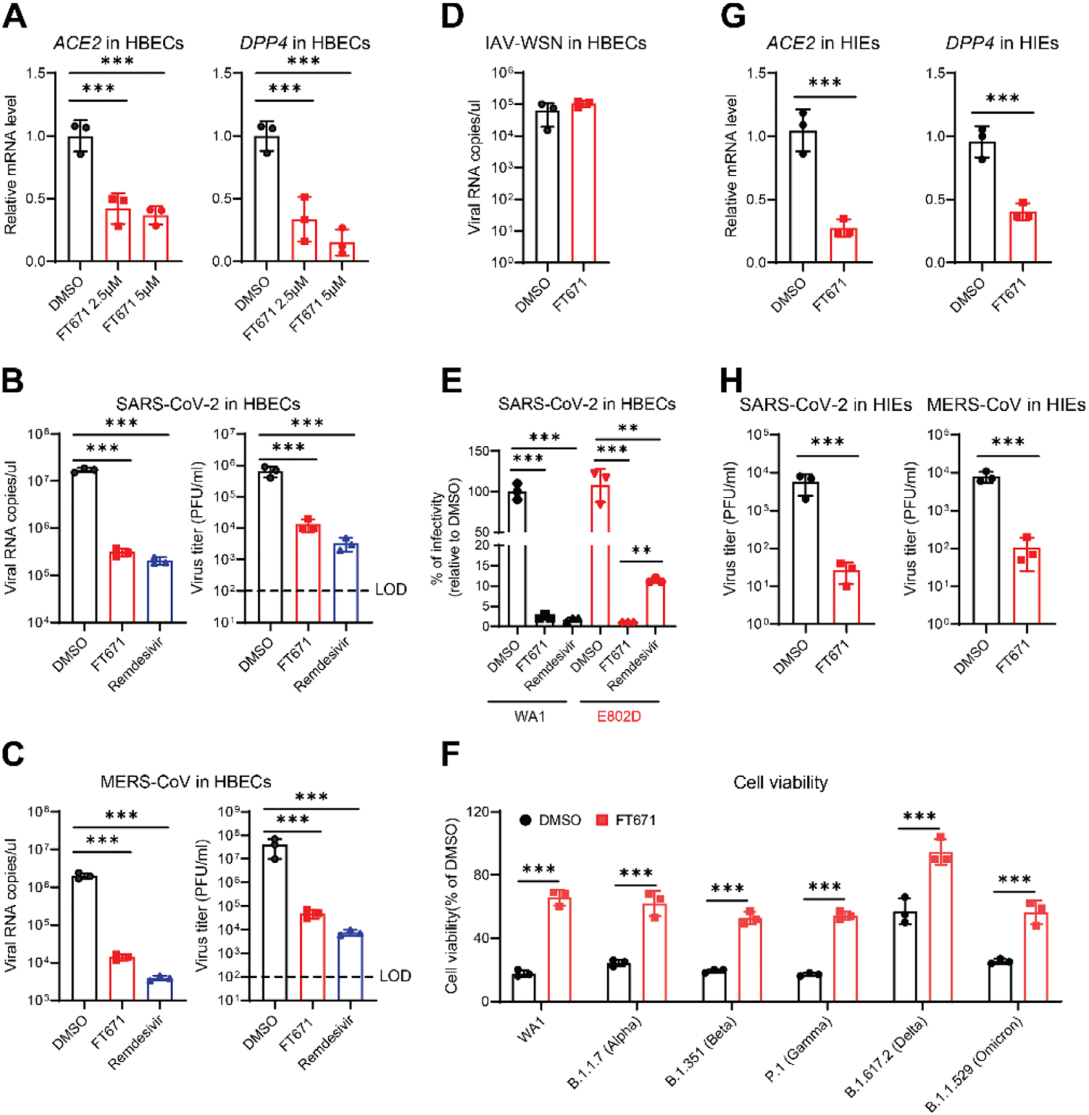
Inhibition of USP7 reduces ACE2 and DPP4 expression and blocks viral infection in primary human cells. (A) FT671 inhibits ACE2 and DPP4 expression in HBECs. HBECs were pretreated for FT671 for 2 days before qPCR experiments. n = 3. (B‐D) Effects of FT671 on viral infection. HBECs were pretreated with FT671 for 2 days, then infected with SARS‐CoV‐2(B), MERS‐CoV (C) or IAV‐WSN (D). Virus replication and virus production were measured at 2 dpi by qPCR and plaque assay, respectively. n = 3. Dash line, limit of detection. (E) Effects of FT671 and Remdesivir on SARS‐CoV‐2 WA1 and E802D strains replication in HBECs. HBECs were pretreated with FT671 or Remdesivir for 2 days, then infected with SARS‐CoV2 or E802D. Virus production was measured by plaque assays. n = 3. (F) FT671 inhibits diverse variants of SARS‐CoV‐2. Vero E6 cells were pretreated with FT671 for 2 days, then infected with the indicated SARS‐CoV2 variants at an MOI of 0.2. Cell viability was measured at 3 dpi. n = 3. (G) FT671 inhibits ACE2 and DPP4 mRNA levels in HIEs. HIEs were treated with FT671 for 2 days before qPCR experiments. n = 3. (H) FT671 inhibits viral infection in HIEs. HIEs were pretreated with FT671 for 2 days, then infected with SARS‐CoV‐2 or MERS‐CoV at 10^5^ PFU/ml for 2 days. Virus production was measured by plaque assays. n = 3. The bar graphs show the mean ± SD of three independent experiments. n, number of independent experiments; Unpaired Student's t test for (D, F, G, and H); One‐way ANOVA with Tukey's test for (A‐C and E); ^∗∗∗^ *p* < 0.001.

Next, we sought to evaluate the antiviral efficacy of USP7 inhibition in physiologically relevant cells. We first determined the impact of USP7 inhibition in primary human bronchial epithelial cells (HBECs) cultured at the air‐liquid interface. Consistent with our findings in cell lines, USP7 inhibition reduced ACE2 and DPP4 expression in primary human airway cells (Figure 7A). FT671 treatment inhibited SARS‐CoV‐2 and MERS‐CoV viral replication and virus production in HBECs as measured by qPCR and plaque assay, respectively (Figure 7B,C). However, influenza A virus (IAV) replication was not affected in FT671‐treated HBECs (Figure 7D), suggesting a degree of viral specificity of the USP7 inhibitor in primary human airway cells.

We next asked whether inhibition of USP7 could inhibit diverse SARS‐CoV‐2 variants of concern and virus resistant to a direct‐acting antiviral. We determined the efficacy on drug‐resistant virus infection using a remdesivir‐resistant SARS‐CoV‐2, which contains a point mutation, E802D, in the RNA‐dependent RNA polymerase (RdRp) [7]. Notably, FT671 exhibited better efficacy against E802D‐mutant virus compared to remdesivir in HBECs (Figure 7E). We observed similar anti‐viral effects of FT671 on diverse SARS‐CoV‐2 variants, including alpha (B.1.17), Beta (B.1.351), Gamma (P.1), Delta (B.1.617.2), and Omicron (B.1.1.529) (Figure 7F). Finally, we tested the USP7 inhibitor efficacy in primary human intestinal enteroids (HIEs), which are permissive for SARS‐CoV‐2 and MERS‐CoV. Pretreatment of FT671 inhibitor reduced ACE2 and DPP4 expression and viral infection in HIEs in 3D culture (Figure 7G,H). Taken together, these data suggest that USP7‐mediated KDM6A homeostasis is critical for ACE2 and DPP4 expression and virus infection in primary human cells.

### 2.8 Inhibition of USP7 Reduces KDM6A Expression and Viral Infection in Mice

**FIGURE 8:**
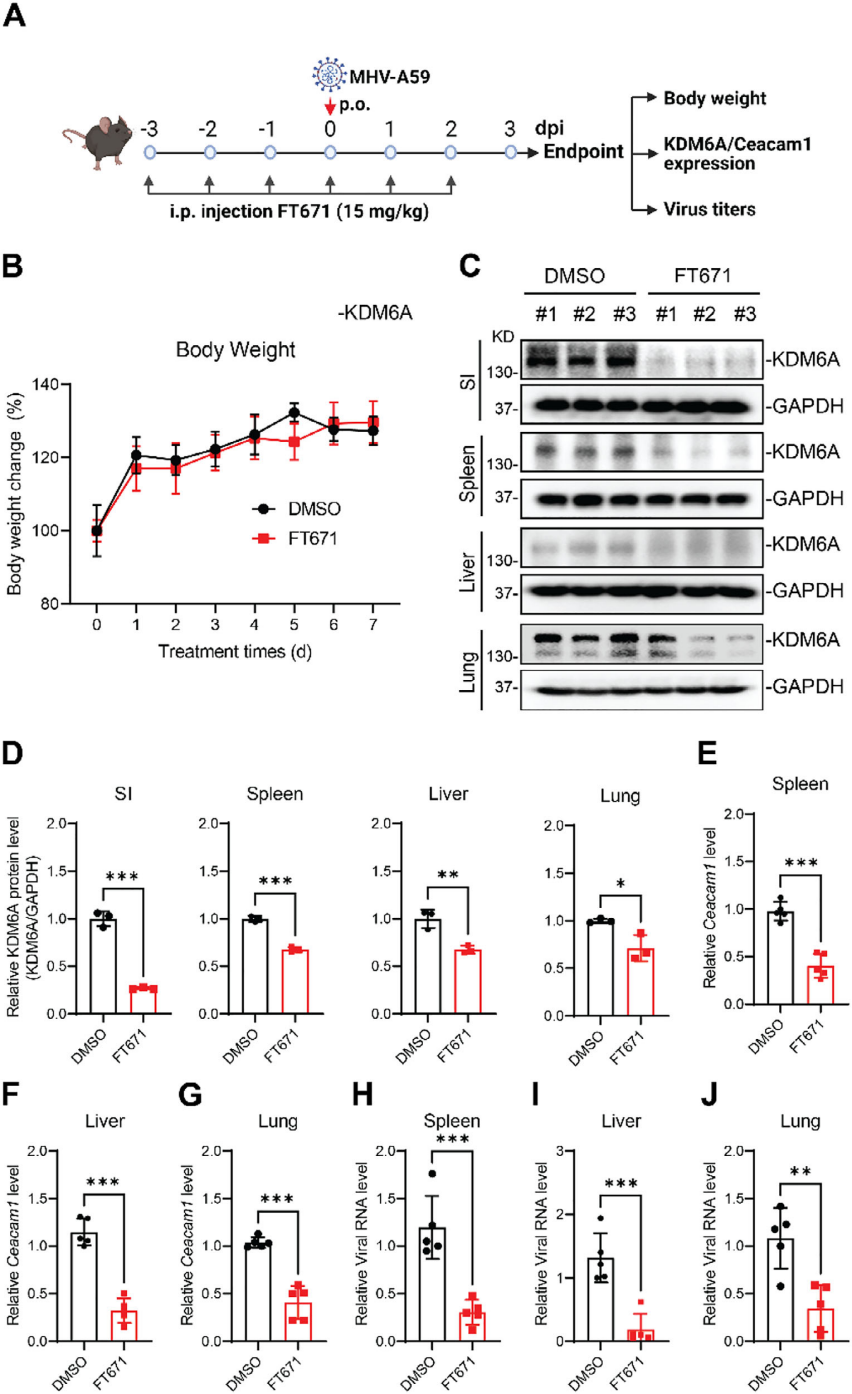
Inhibition of USP7 reduces KDM6A expression and viral infection in mice. (A) Schematic of FT671 USP7 inhibitor treatment (i.p. daily at 15 mg/kg) and virus infection in C57BL/6 J mice, timeline indicated for days post infection (dpi). (B) Body weight change of mice injected with FT671 or DMSO. The C57BL/6J mice were i.p. injected with DMSO or FF671 at 15 mg/kg for 5 days, body weight was monitored every day. n = 5 mice. (C‐D) Effects of FT671 on KDM6A expression in mice. C57BL/6J mice were i.p. injected with DMSO or FT671 at 15 mg/kg for 5 days, and the small intestine, spleen, liver, and lung were collected to measure KDM6A expression by western blot (C) and quantified the protein abundance (D). n = 3 mice. (E‐G) Effects of FT671 on viral receptor Ceacam1 expression. The C57BL/6J mice were i.p. injected with DMSO or FT671 at 15 mg/kg for 5 days, the spleen (E), liver (F), and lung (G) tissues were collected to measure Ceacam1 mRNA level by qPCR analysis. n = 5 mice. (H‐J). Effects of FT671 on MHV‐A59 infection in mice. The C57BL/6J mice were i.p. injected with DMSO or FT671 at 15 mg/kg for 3 days, the mice were infected by oral gavage with 1 × 10^5^ PFU MHV‐A59 per mouse. The mice continued to be injected with FT671 for another 2 days. At 3 dpi, spleen (H), liver (I), and lung (J) tissues were collected to measure viral burden by qPCR analysis. n = 5 mice. The bar graphs show the mean ± SD of the indicated number of mice. n, number of mice; Unpaired Student's t test for (D to J); ∗ p < 0.05; ∗∗ p < 0.01; ∗∗∗ p < 0.001.

Finally, we investigated the therapeutic potential of USP7 inhibition in vivo in mice (Figure 8A). We first evaluated the toxicity of USP7 inhibitor treatment in C57BL/6J mice. We did not observe significant body weight loss in FT671‐treated mice compared to DMSO control, demonstrating tolerance of USP7 inhibition in mice (Figure 8B). Given the role of USP7 in maintaining KDM6A stability, we determined the impact of FT671 treatment on KDM6A protein expression in mice. As expected, FT671 treatment decreased KDM6A protein level in small intestine, spleen, liver, and lung tissues (Figure 8C,D). Next, we examined the impact of FT671 treatment on mouse hepatitis virus receptor expression. FT671 treatment reduced viral receptor Ceacam1 expression in different tissues, including spleen, liver, and lung (Figure 8E–G). Consistent with the reduced viral receptor expression, FT671 treatment of mice conferred protection from MHV‐A59 infection as measured by virus replication in the spleen, liver, and lung tissues (Figure 8H–J). Collectively, these data demonstrate the tolerability and antiviral efficacy of USP7 inhibition in mice.

## 3 Discussion

Our study reveals a finely tuned, ubiquitin‐mediated regulatory circuit centered on KDM6A stability that dictates cellular susceptibility to multiple coronaviruses. We demonstrate that the deubiquitinase USP7 stabilizes KDM6A by removing K48‐linked polyubiquitin chains, thereby upregulating the expression of multiple coronavirus receptors and promoting viral entry. Conversely, the E3 ubiquitin ligase RNF40 antagonizes this process by catalyzing K6‐ and K11‐linked ubiquitination of KDM6A, which recruits the selective autophagy receptor TAX1BP1 to trigger autophagic degradation of KDM6A and restrict viral infection. Genetic and pharmacological inhibition of USP7 substantially reduces viral receptor expression and blocks infection in cell lines, primary human airway epithelial cells, and mouse models, highlighting the therapeutic potential of targeting USP7 against current and emerging coronaviruses.

The existence of this intricate antagonistic switch‐USP7‐mediated stabilization versus RNF40/TAX1BP1‐mediated autophagic degradation‐underscores the critical need for cells to dynamically and precisely control KDM6A protein levels. Beyond its role in coronavirus receptor expression, KDM6A, as a master epigenetic regulator of developmental and immune genes [49, 50]. must be maintained within an optimal range to ensure proper cellular function. Excessive KDM6A could lead to the sustained opening of chromatin and overexpression of genes involved in inflammatory responses, potentially triggering immunopathology or autoimmunity. Conversely, insufficient KDM6A might cripple the transcriptional plasticity required for mounting effective innate and adaptive immune defenses [51, 52]. Therefore, the USP7/RNF40 axis may serve as a crucial homeostat, allowing the host to rapidly tune epigenetic landscapes in response to infections like coronaviruses. By degrading KDM6A via RNF40, the cell can potentially dampen excessive inflammation and limit viral spread, while the USP7‐mediated stabilization might facilitate the expression of genes necessary for tissue repair and immune memory once the acute threat subsides. This fine‐tuning underscores how ubiquitin‐dependent control of an epigenetic modifier is co‐opted to maintain immune balance during viral challenge.

While our study focuses on the role of USP7 in stabilizing KDM6A to promote viral entry, emerging evidence suggests USP7 may have broader functions in antiviral defense. In our ubiquitination assays indicated ubiquitination of KDM6A was not completed reversed upon ectopic expression of USP7‐C223S, suggesting that other DUB(s) may compensate for the loss of USP7 catalytic activity. USP7 has been shown to regulate diverse immune signaling pathways, potentially affecting viral replication through multiple mechanisms beyond receptor expression [53]. For instance, USP7 can modulate type I interferon responses and NF‐κB signaling by stabilizing key immune regulators [54, 55]. These suggest that USP7 inhibition might exert antiviral effects through both receptor‐dependent and receptor‐independent mechanisms. Notably, our observation that USP7 inhibition did not affect influenza A virus replication, suggesting a degree of specificity for coronaviruses that warrants further investigation.

Beyond the USP7‐mediated classical proteasomal degradation of KDM6A, our identification of RNF40‐driven, TAX1BP1‐mediated autophagic degradation of KDM6A represents a conceptual advance in the field of virus‐host interactions. Autophagy has long been recognized as a double‐edged sword in viral infection, with some viruses exploiting autophagy components for replication while others are restricted by autophagic degradation [56, 57]. Our finding that RNF40‐mediated K6/K11‐linked ubiquitination serves as a recognition signal for TAX1BP1‐dependent autophagy provides a paradigm for understanding how epigenetic regulators can be targeted for selective autophagy. Specifically, our data demonstrate that USP7 stabilizes KDM6A by removing proteasome‐targeting K48‐linked ubiquitin chains, while RNF40 promotes its TAX1BP1‐mediated autophagic degradation via K6/K11‐linked ubiquitination. This dual regulation through distinct ubiquitin linkages exemplifies an intricate layer of control over epigenetic regulators, expanding the functional repertoire of non‐proteolytic ubiquitination in modulating viral infection. Although USP7 removes K48‐linked polyubiquitin chains from KDM6A, it does not counter RNF40‐mediated K6‐ and K11‐linked polyubiquitination, underscoring the specificity of E3 ligases in catalyzing distinct ubiquitin chain linkages. These findings highlight the need to identify additional E3 ubiquitin ligases and DUBs that regulate KDM6A and to evaluate their roles in relevant physiological processes.

Our findings establish USP7 and RNF40 as critical upstream regulators of KDM6A stability. This regulatory axis is likely involved in a broader cellular network, as both USP7 and RNF40 are themselves subject to stringent regulation, potentially forming a more complex regulatory circuit. Transcriptionally, the expression of USP7 is regulated by multiple transcription factors including NOTCH1, FOXO6, and STAT3 in certain contexts [58]. Similarly, RNF40 expression is regulated at the RNA level by m6A methylation, influencing histone ubiquitination and gene expression program [59]. At the post‐translational level, both enzymes are tightly regulated. USP7 activity and stability can be modulated by modifications such as phosphorylation and UFMylation [60]. and it can even undergo autodeubiquitination [61]. creating feedback loops that fine‐tune its function. RNF40, as part of complexes with RNF20 or other partners, can be regulated by phosphorylation events that affect its E3 ligase activity and substrate specificity [62]. This reciprocal regulation creates a highly responsive and adaptable network, allowing cells to precisely balance KDM6A homeostasis in response to diverse intracellular and extracellular signals, including viral infection. Future studies investigating how viral infection or immune signaling affect the expression and activity of USP7 and RNF40 will be crucial to fully understand the dynamics of this regulatory loop.

Genes encoding USP7 and KDM6A are frequently mutated in developmental disorders and cancers [34, 63, 64]. Previous studies have shown that the demethylase activity of KDM6A is dispensable for tumor suppression, enhancer regulation, and viral receptor expression [38, 65, 66]. Our data show that the ubiquitination and homeostasis of KDM6A mediated by USP7 and RNF40 are critical for the expression of ACE2, DPP4, and Ceacam1 and for viral infection. Previous studies have also shown that USP7 and KDM6A are significantly upregulated in multiple cancers and their increased levels correlate with more aggressive tumors and poor prognosis [31, 32, 34]. Therefore, exploring the physiological significance of the interplay between USP7 and KDM6A in cancers and developmental disorders represents an important future direction.

Recent work from our group and others has identified numerous host factors that modulate ACE2 expression, including transcription factors, epigenetic regulators, and signaling pathways, underscoring the multi‐layered regulatory landscape governing viral receptor expression [22, 23, 24, 26, 38, 67]. Epigenetic regulators such as the BAF complex, HMGB1, DYRK1A, and the KDM6A‐KMT2D‐p300 axis mediate chromatin accessibility and enhancer activity [22, 23, 38]. Transcription factors, including HNF1A/B and GATA6, bind to the *ACE2* locus to initiate transcription [23, 35]. while suppression of FXR signaling and BRD2 diminishes ACE2 transcriptional expression and restricts SARS‐CoV‐2 infection [24, 26]. Here, we demonstrate that USP7 and RNF40 regulate ACE2 expression and SARS‐CoV‐2 infection by controlling KDM6A ubiquitination. Importantly, this axis also governs DPP4 and Ceacam1 expression, thereby influencing MERS‐CoV and MHV infection. Pharmacological inhibition of the USP7‐KDM6A axis exhibited pan‐coronavirus antiviral activity against diverse coronaviruses in vitro and in vivo, further supporting the targeting of this axis as a viable host‐directed strategy against coronavirus infections. Unlike virus‐directed therapies, host‐directed approaches targeting USP7 are less susceptible to viral mutation and escape, offering a durable strategy against emerging coronaviruses. Despite the proviral role of the USP7‐KDM6A axis in coronavirus infection, both molecules exhibit tumor‐suppressive functions in certain cancers [34, 63, 64]. This functional duality implies that therapeutic targeting of this axis would require careful evaluation of tissue‐specific effects and potential long‐term consequences using tissue‐specific KO mice. The restricted expression pattern of coronavirus receptors in specific tissues may provide a therapeutic window wherein USP7 inhibition could block viral entry without broadly disrupting physiological functions. The favorable tolerability of FT671 in mice and its efficacy in human primary airway and intestinal epithelial cells further highlights its therapeutic potential. Future studies should prioritize a rigorous assessment of potential adverse effects arising from the inhibition of USP7 and KDM6A, while exploring optimal dosing and combination strategies with direct‐acting antivirals to maximize efficacy and mitigate risks.

In conclusion, our findings unveil a finely balanced, ubiquitin‐mediated circuit that controls KDM6A stability and governs cellular permissiveness to diverse coronaviruses. Targeting this axis, particularly through inhibition of USP7, represents a promising broad‐spectrum strategy against current and future coronavirus threats.

## 4 Experimental Section

### 4.1 Cells

Vero E6 (CRL‐1586), HEK293T (CRL‐3216) were purchased from ATCC; Vero‐E6‐ACE2‐TMPRSS2 (NR‐54970) and Calu‐3 2B4 (NR‐55340) were obtained from BEI resources. Huh7.5 was a gift from Dr Charlie Rice (Rockefeller); Primary human bronchial epithelial cells (HBECs) (CC‐2540) were purchased from Lonza. Human intestinal enteroids (J2) were obtained from Dr Mary Estes (Baylor College of Medicine). Vero E6, Vero‐E6‐ACE2‐TMPRRS2, Huh7.5, and HEK293T cells were cultured in DMEM medium containing 10% FBS and 1% Penicillin/Streptomycin. Calu‐3 cells were cultured in RPMI 1640 medium with 10% FBS, 1% Penicillin/Streptomycin, and Glutamax. HBECs were cultured in air‐liquid interface in transwell according to manufacturer's protocol (STEMCELL Technologies). Protocol for culture and infection of HBECs and HIEs was described as previously [23, 68].

### 4.2 Expression Constructs

The following constructs were obtained from Addgene: pQFlag‐USP7 WT (46751), pQFlag‐USP7 C223S (46752), HA‐Ubi (17608), HA‐Ubi‐K48O (17604), plentiCRISPR‐V2 (52961), pLX307‐HNF1B‐puro (98342), EF1a‐HNF1A‐p2a‐Hygro (120448). pInducer20‐KDM6A was provided by Dr Hao Jiang (University of Virginia); HA‐Ubi‐K6O, HA‐Ubi‐K11O, HA‐Ubi‐K27O, HA‐Ubi‐K33O were provided by Dr Hongbing Shu (Wuhan University); TOLLIP‐HA, NIX‐HA, NDP52‐HA, TAX1BP1, OPTN‐HA, and P62‐HA were provided by Dr Wenrui He (Henan Agricultural University).

pCAGSS‐Stag‐KDM6A, RNF40, RNF20, and KCMF1 were generated by inserting PCR fragments of corresponding gene into pCAGSS‐Stag vector. pCAGSS‐Stag‐KDM6A K29R, K299R, K1315R, K29/1315R, K299/1315R, K29/299/1315R and PRK‐HA‐Ubi K6/K11O, K6/K11R were generated by PCR‐based site directed mutagenesis. PLX304‐V5‐HNF1A, PLX304‐V5‐HNF1B, pLX304‐F‐RNF40, pLX304‐F‐RNF40‐ΔRING, pLX304‐V5‐KDM6A WT and K29/299/1315R were generated by amplifying the corresponding gene from the template plasmids and cloning into pLX304‐V5‐Blast.

### 4.3 Viruses

The following viruses were used in this study were produced as described previously [22, 23, 38]. SARS‐CoV‐2 (NR‐52281), HKU5‐SARS‐CoV‐1‐S (NR‐48814), B.1.1.298 (NR‐53953), B.1.1.7 (NR‐54000), B.1.351 (NR‐54008), P.1 (NR‐54982), B.1.617.2 (NR‐55611), MERS‐CoV (NR‐48811), MHV‐A59 (NR‐4300) from BEI resources, Omicron from Yale New Haven Hospital. icSARS‐CoV‐2‐mNG reporter virus was from the World Reference Center for Emerging Viruses and Arboviruses (Galveston, TX). All work with infectious viruses was performed in a Biosafety Level 3 (BSL3) laboratory and approved by the Yale University Biosafety Committee. MHV‐3 was obtained from ATCC. MHV‐A59 was provided by Dr Yang Qiu (Wuhan Institute of Virology). All MHV infection experiments were performed in a BSL2 laboratory.

### 4.4 Plaque Assay

Plaque assays were described as previously [22, 23]. In brief, either Vero E6 or Vero E6‐ACE2‐TMPRSS2 cells for human coronavirus and L2 cells for MHV were seeded in 12‐well plates at 4 × 10^5^ cells per well. The following day, the media was removed and replaced with 100 µl of tenfold serial dilutions of the virus. Plates were incubated at 37°C for 1 h with gentle rocking every 10 min. Subsequently, the media were replaced with overlay media. At 2–3 dpi, remove the overly media and fix cells with 10% formaldehyde for 30 min before visualizing with 0.5% crystal violet solution.

### 4.5 Generation of Polyclonal KO Cell Lines

CRISPR sgRNA target sequences were cloned into the lentiCRISPR‐V2 and verified by Sanger sequencing. To prepare lentiviral particles, supernatants were collected from HEK293T cells co‐transfected sgRNA and packaging plasmids (psPAX2 and VSVG). The target cells were transduced with lentiviral particles and puromycin or G418 was added 2 days later. Cells were selected with puromycin or G418 for 5–7 days before additional experiments were conducted. The corresponding sgRNA target sequences used in this study are shown in Table S1.

### 4.6 Generation of USP7 and RNF40 Knockout and Complemented Cells

Huh7.5 USP7 and RNF40 KO cells were generated by the transfection of Cas9‐RNPs. Single cells were seeded in 96‐well plate and USP7 knockout efficiency was confirmed by western blot. USP7 KO clones were reconstituted by retroviral transduction of pQFlag‐puro empty vector or containing human wild‐type USP7, a deubiquitinase inactive USP7 mutant (C223S). RNF40 KO cells were reconstituted by lentiviral transduction of pLX304‐blast empty vector or containing wild‐type KDM6A, the enzyme‐inactive mutant (ΔRING). Twenty‐four hours post transduction, puromycin or blasticitin was added and cells were selected for seven days. The USP7 and RNF40 expression was confirmed by western blot in complemented cells.

### 4.7 USP7 Inhibitor Treatment for Cell Lines

FT671 (HY‐107985) and XL177A (HY‐138794) were purchased from Med Chem Express. Vero E6, Huh7.5, and Calu3 cells were pretreated with FT671 for the indicated times and then infected with virus at 0.1–0.5 MOI. Cell viabilities were measured by CellTiter Glo at 2–3 days post infection. Viral receptors ACE2 and DPP4, Ceacam1 expressions were measured by qPCR or western blot following FT671 pretreatment. HBECs and HIEs were pretreated with FT671 for 2 days and then infected with virus at MOI = 0.5 or 5 × 10^6^ PFU/ml, the virus replication and virus production were measured by qPCR and plaque assays, respectively.

### 4.8 USP7 Inhibitor Treatment and MHV‐A59 Infection in Mice

C57BL/6J mice were injected intraperitoneally (i.p.) daily with FT671 (15 mg/kg) for 5 d. For FT671 cytotoxicity assays, body weight was monitored daily following FT671 treatment. For KDM6A protein expression, tissues were collected and homogenized in 1 mL lysis buffer, then 250 µl of homogenate was mixed with SDS loading buffer before western blot analysis. For Ceacam1 expression, tissues were collected and homogenized in 1 mL of DMEM with 1% penicillin‐streptomycin. Then 500 µl of homogenate was mixed with 500 µl of TRIzol, and RNA was extracted for Ceacam1 qPCR analysis. For infections, mice were anesthetized with 30% isoflurane and administered MHV‐A59 by oral gavage (p.o.) in 50 µl PBS. At 3 dpi, the lung, liver, spleen were collected and homogenized in 1 mL DMEM with 1% penicillin‐streptomycin. Tissue homogenates were mixed with TRIzol and RNA extracted for viral replication by qPCR analysis. All work with mice was performed in the animal BSL‐2 facility with approval from the Institutional Animal Care and Use Committee at Wuhan Institute of Virology (Ethical Approval Number: WIVA37202501). Mice were randomized based on sex for these experiments.

### 4.9 Pseudovirus Assays

293T cells were transfected by corresponding CoV spike (S) glycoproteins and then inoculated with a VSV seed particle virus that contains Renilla luciferase or GFP instead of the VSV‐G. The cells were washed with PBS to remove the inoculum at one hour post‐infection. After an incubation of 24 h, supernatant containing pseudotyped virus was collected and stored at −80°C before use.

Vero E6, Huh7.5 or BV2 cells were seeded in 96‐well plates and incubated overnight. The next day pseudotyped virus was added at 1:10 volume/volume and incubated for 24 h. Then cells were lysed with passive lysis buffer (Promega) at room temperature before luciferase activity was measured using a BioTek plate reader.

### 4.10 RT‐qPCR

Total RNA was isolated from Huh7.5, Vero E6, Calu‐3, HBECs, and HIEs Cells by the standard Trizol RNA extraction protocol according to the ZYMO RNA MiniPrep Plus kit manufacturer's instructions. The extracted RNA was reverse transcribed to cDNA using MLV with random primers. Gene expressions were quantified by qPCR using specific primers that are shown in Table S2. All qPCR results were obtained using QuantStudio 1 plus or QuantStudio 3.

### 4.11 ChIP‐qPCR

KDM6A and H3K27ac ChIPs were performed using ChIP‐IT High Sensitivity kit following the manufacturer's instructions. Briefly, WT or USP7 KO Huh7.5 cells (40 × 10^6^/per assay) were fixed initially with 25 mM DMA at room temperature for 1 h, and subsequently with 1% formaldehyde at room temperature for 10 min. Quench the formaldehyde by adding Glycine diluted to a final concentration of 125 mM at room temperature for 5 min. Fixed cells were washed twice with PBS, and the cell pellet was collected and stored at −80°C before ChIP assays. The fixed cells were lysed with the provided lysis solution supplemented with protease inhibitors. Next, chromatin was sonicated to obtain DNA fragments within the recommended 200–500 bp range. In total, 100 µg of sheared chromatin was then incubated with 2 µg of antibody against KDM6A, or H3K27ac, overnight at 4°C with rotation. Following incubation with Protein G agarose beads, bound chromatin was washed, eluted, and purified following the manufacturer's protocols. ChIP DNAs were analyzed by qPCR. Data were normalized to the percentage of input DNA. ACE2 promoter and enhancer qPCR primers are shown in Table S2.

### 4.12 Coimmunoprecipitation

Cells were scrambled off from dishes and lysed in NP‐40 or RIPA lysis buffer. The cells were sonicated and centrifuged to clarify cell debris. Then 0.5‐ml aliquot of lysate was incubated with the indicated antibodies or IgG isotype and Protein‐G for at least 2 h. The precipitates were washed at least three times with NP‐40 lysis buffer. For each immunoprecipitation, 50 µl SDS loading buffers were added to the precipitates and boiled at 100°C for 10 min. The precipitates were used for western blot.

### 4.13 In Vitro Binding Assays

The HA‐Ubi‐K6/K11O, K6/K11R or WT ubiquitin conjugated KDM6A were purified from HEK293T cells transfected with KDM6A‐V5 together with HA‐Ubi WT, K6/K11O or K6/K11R mutants. The transfected cells were collected and lysed in NP‐40 lysis buffer, then cell lysates were incubated with V5 antibody and protein‐G for 3 h. The precipitates were resuspended in new NP‐40 lysis buffer, then subjected with HA antibody pull‐down. The two‐step immunoprecipitated precipitates were used as the indicated purified ubiquitin‐conjugated KDM6A. The TAX1BP1 protein was purified from HEK293T cells transfected with Flag‐TAX1BP1. The purified proteins were incubated at 4°C and immunoprecipitated with Flag antibody for 3 h. The precipitates were used for western blot.

### 4.14 Western Blot

Co‐IP precipitates and cell lysates were fractionated on SDS‐PAGE and transferred onto polyvinylidene fluoride (PVDF) membrane (Millipore). Antibodies used in this study are listed in Table S3. Immunoblotting analysis was performed following standard method with the indicated antibodies and visualized with HRP‐conjugated goat anti‐Rabbit or goat anti‐Mouse IgG by chemiluminescence detection system (Invitrogen iBright).

### 4.15 Statistical Analysis

All statistical analyses were performed using GraphPad Prism Version 9. A two‐tailed unpaired t‐test was used for comparisons between two groups, and one‐way analysis of variance (ANOVA) with Tukey's test was used for comparisons involving more than two conditions. All data are shown as the mean ± standard deviation. n, represents the number of independent experiments. For in vivo data, n represents the number of mice. In all analyses, 95% confidence intervals (95% CI) were calculated, and *P*‐values less than 0.05 were considered statistically significant. All statistically *p*‐value is represented by a symbol (^*^ *p* <0.05, ^**^ *p* <0.01, ^***^ *p* <0.001). The absence of a bar indicated no statistical pairwise comparisons were made. All *P‐*values were listed in Table S4.

## Author Contributions

L.L. and J.W. conceived and designed the study. M.Z.H., Z.Y.Y., S.W., Y.G., L.L., M.M.A., R.F., M.Z.K., W.Y.S., and J.W. performed experiments and analyzed the data. J.W. provided resources and funding acquisition. J.W. supervised the study. J.W. wrote the manuscript with input from all authors. All authors have reviewed and edited the manuscript.

## Conflicts of Interest

The authors declare no conflicts of interest.

## Supporting information




**Supporting File 1**: advs74399‐sup‐0001‐SuppMat.docx.

## Data Availability

The data that support the findings of this study are available from the corresponding author upon reasonable request.
