# Supplementary material for: Antagonistic Ubiquitin Switching by USP7 and RNF40 Orchestrates KDM6A Homeostasis to License Coronavirus Susceptibility
Source: Adv Sci (Weinh). 2026 Feb 15;13(23):e18058. doi: 10.1002/advs.202518058 (PMC13104090; doi:10.1002/advs.202518058)
Supplement: Supplementary file 1 — Supporting File 1: advs74399‐sup‐0001‐SuppMat.docx. [file ADVS-13-e18058-s001.docx]

Supporting Information

**Antagonistic Ubiquitin Switching by USP7 and RNF40 Orchestrates KDM6A Homeostasis to License Coronavirus Susceptibility**

*Meng-Zhuo Huang, Zhong-Yuan Yang, Shi Wang, Yan Ge, Lan Lin, Mia Madel Alfajaro, Renata B. Filler, Wan-Yao Song, Ming-Zhu Kong, Jin Wei**

1. Figures


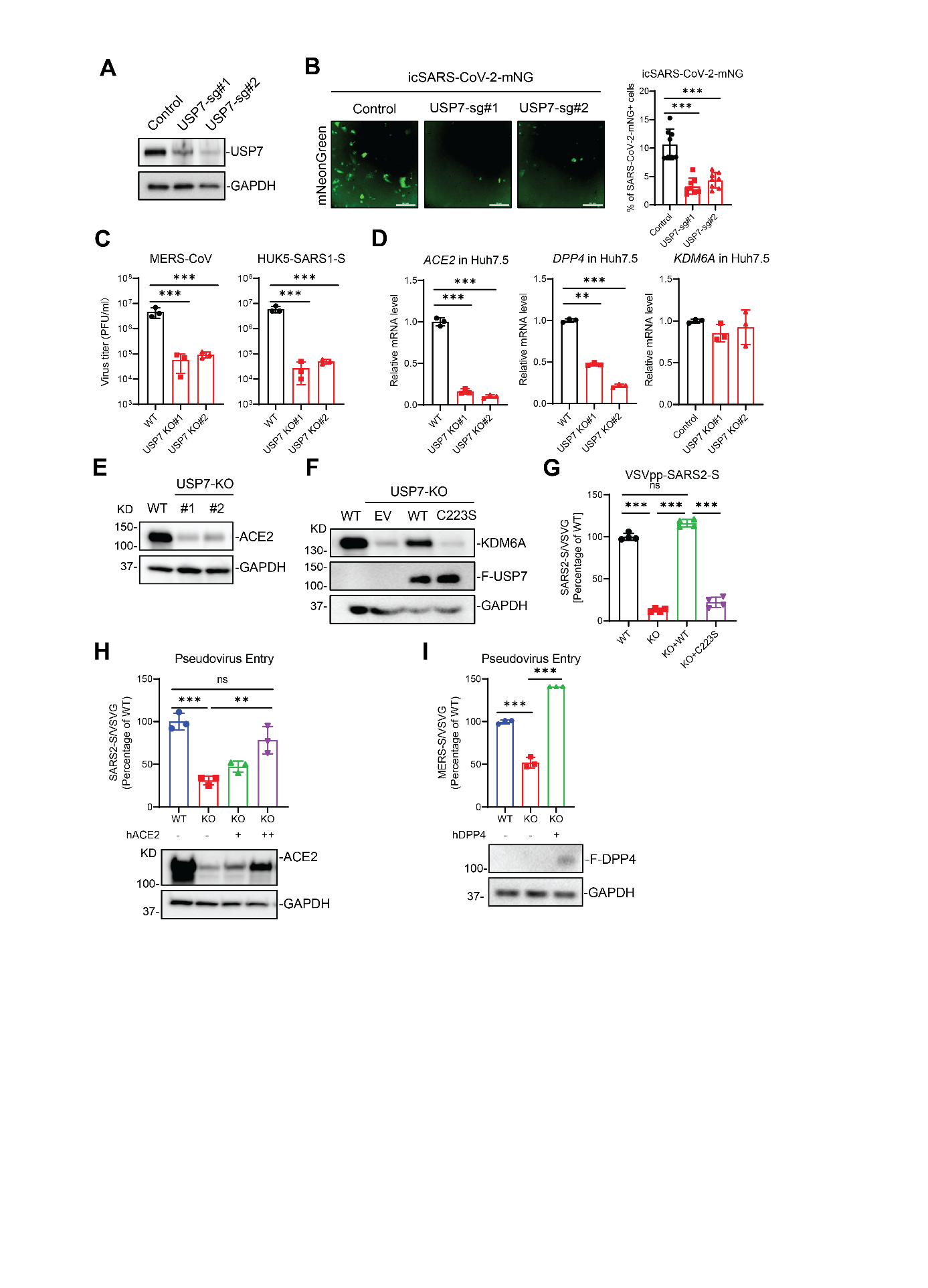


**Figure S1.** USP7 deficiency inhibits SARS-CoV-2 infection. (A) CRISPR-mediated USP7 polyclonal KO was confirmed by Western blot in Vero E6 cells. (B) Viral replication in USP7 polyclonal KO Vero E6 cells. Cells were infected with icSARS-CoV-2-mNG at an MOI of 1. Infected cells were imaged via fluorescence microscopy (left) and mNeonGreen expressing cell frequency was measured 2 dpi (right). n=8. Scale bar: 300 μm. (C) Viral production in WT and USP7 KO Huh7.5 cells. Cells were infected with MERS-CoV pr HKU5-SARS1-S at a MOI of 0.1, then the virus production was measured by plaque assays in Vero E6 cells. n=3. (D) Gene expression in USP7 KO clones was measured by qPCR in Huh7.5 cells. n=3. (E) ACE2 expression in USP7 KO clones was determined by Western blot. (F) USP7 expression in USP7 KO Huh7.5 cells reconstituted with EV, USP7 WT and C223S mutant was determined by western blot. (G) USP7 KO cells were infected with VSVpp-SARS2-GFP pseudovirus. Infected cells were imaged via fluorescence microscopy and GFP expressing cell frequency was measured 1 dpi. n=4. (H-I) Overexpression of human ACE2 (H) and DPP4 (I) in USP7 KO Huh7.5 cells and infected the cells with SARS-CoV-2 and MERS-CoV-2 pseudoviruses respectively. n=3. The bar graphs show the mean ± SD of the indicated independent experiments. n, number of independent experiments; One‐way ANOVA with Tukey’s test for (B to D and G to I); * p < 0.05; ** p < 0.01; *** p < 0.001; ns, not significant.


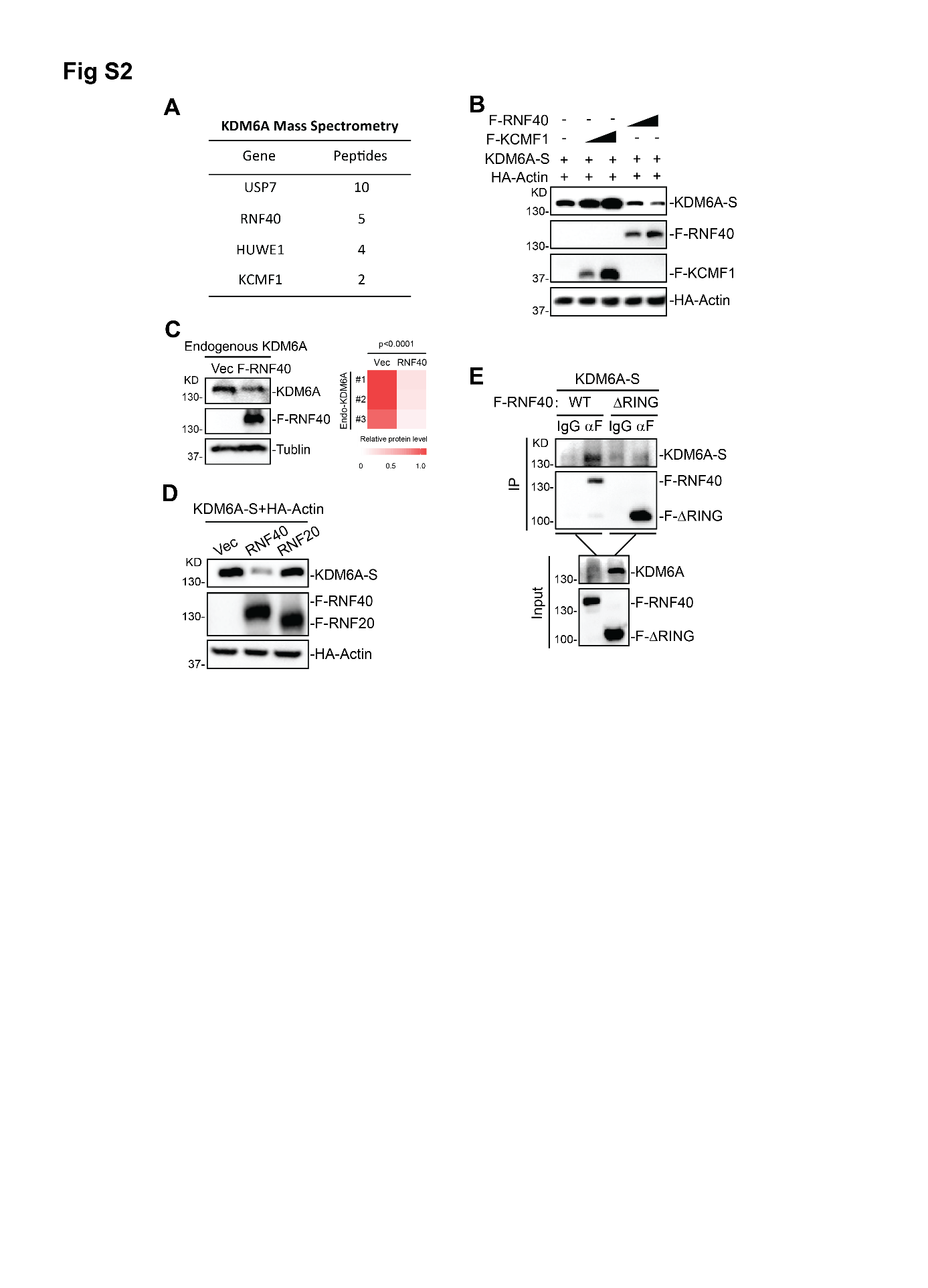


**Figure S2.** RNF40 interacts with KDM6A. (A) Mass spectrometry analysis to identify KDM6A associated E3 ligases in CWR22Rv1 cells. (B) Effects of overexpression of E3 ligases on KDM6A degradation. 293T cells were transfected with KDM6A-S and HA-actin together with increased F-RNF40 or F-KCMF1 for 24 h before immunoblot analysis. (C) Effects of overexpression of RNF40 on endogenous KDM6A expression. 293T cells were transfected with empty vector or Flag-RNF40 for 24 h before immunoblot analysis. (D) Overexpression of RNF40, but not RNF20, degrades KDM6A. 293T cells were transfected with the indicated plasmids for 24 h before immunoblot analysis. (E) RNF40 interacts with KDM6A through its RING domain. 293T cells were transfected with KDM6A-S and HA-actin together with empty vector, Flag-RNF40 or DRING mutant for 24h before coimmunoprecipitation and immunoblot analysis.


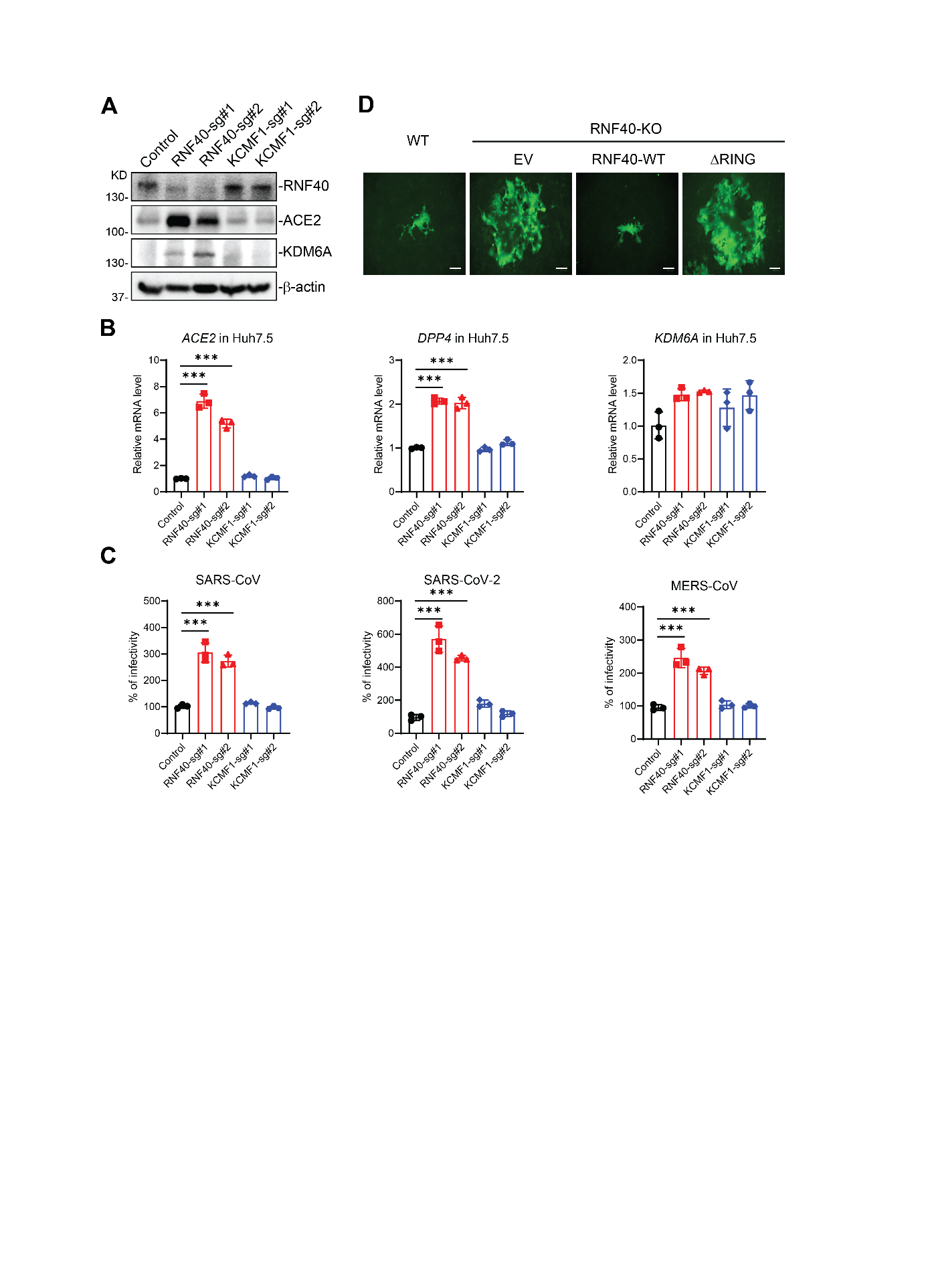


**Figure S3.** RNF40 deficiency promotes viral receptor expression and viral infection. (A) RNF40 deficiency elevated KDM6A protein level. Huh7.5 cells were transduced with RNF40 or KCMF1 sgRNAs by lentiviral-mediated CRISPR-Cas9 gene KO. Cells were selected with puromycin for 5 days before immunoblot analysis. (B) ACE2, DPP4 and KDM6A mRNA expression in RNF40 or KCMF1 polyclonal KO Huh7.5. CRISPR-mediated RNF40 or KCMF1 polyclonal KO cells were processed for qPCR analysis. n=3. (C) Pseudovirus infection in RNF40 or KCMF1 polyclonal KO cells. Cells were infected with VSVpp-GFP pseudotyped SARS-CoV-S, SARS-CoV-2-S and MERS-CoV-S. Infected cells were imaged via fluorescence microscopy and GFP expressing cell frequency was measured 1 dpi. n=3. (D) SARS-CoV-2 Pseudovirus infection in reconstituted RNF40 KO cells. Cells were infected with rcVSV-SARS-CoV-2-S. Infected cells were imaged via fluorescence microscopy at 1 dpi. Scale bar: 100 μm. The bar graphs show the mean ± SD of three independent experiments. n, number of independent experiments; One‐way ANOVA with Tukey’s test for (B and C); *** p < 0.001.


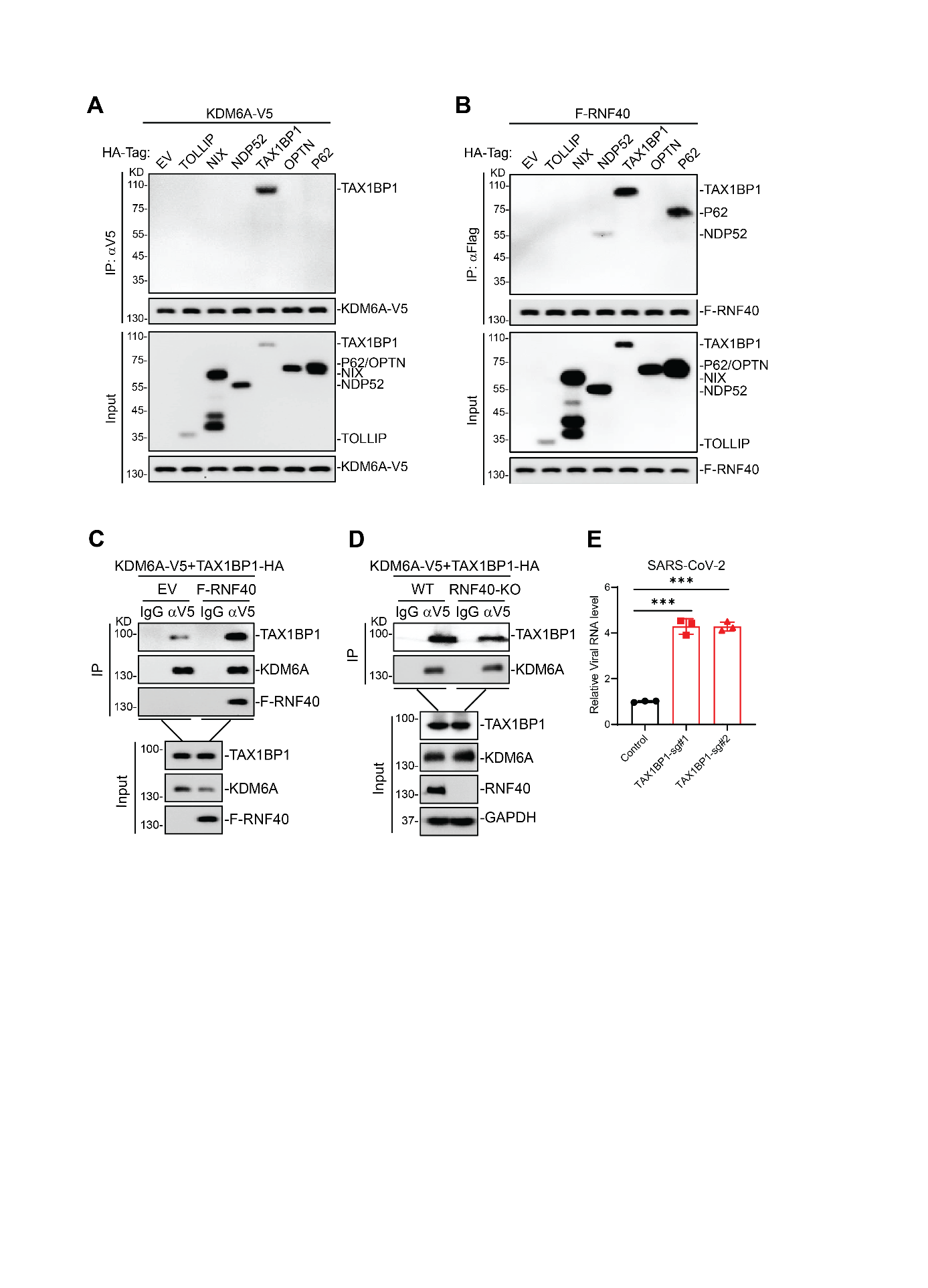


**Figure S4.** RNF40 interacts and promotes the association of TAX1BP1 with KDM6A. (A) KDM6A interacts with TAX1BP1 and p62. 293T cells were transfected with Flag-RNF40 together with the indicated autophagy receptors for 24 h before coimmunoprecipitation and immunoblot assays. (B) RNF40 interacts with TAX1BP1 and p62. 293T cells were transfected with Flag-RNF40 together with the indicated autophagy receptors for 24 h before coimmunoprecipitation and immunoblot assays. (C) RNF40 promotes KDM6A-TAX1BP1 association. 293T cells were transfected with KDM6A-V5 and TAX1BP1-HA together with empty vector or Flag-RNF40 for 24 h before coimmunoprecipitation and immunoblot assays. (D) RNF40 deficiency impairs the KDM6A-TAX1BP1 association. WT and RNF40 KO cells were transfected with the indicated plasmids for 24 h before coimmunoprecipitation and immunoblot analysis. (E) TAX1BP1 deficiency promotes SARS-CoV-2 infection. Cells were infected with SARS-CoV-2 for 1 dpi before Viral RNA level was measured by qPCR. n=3. The bar graphs show the mean ± SD of three independent experiments. n, number of independent experiments; One‐way ANOVA with Tukey’s test for (E); *** p < 0.001.


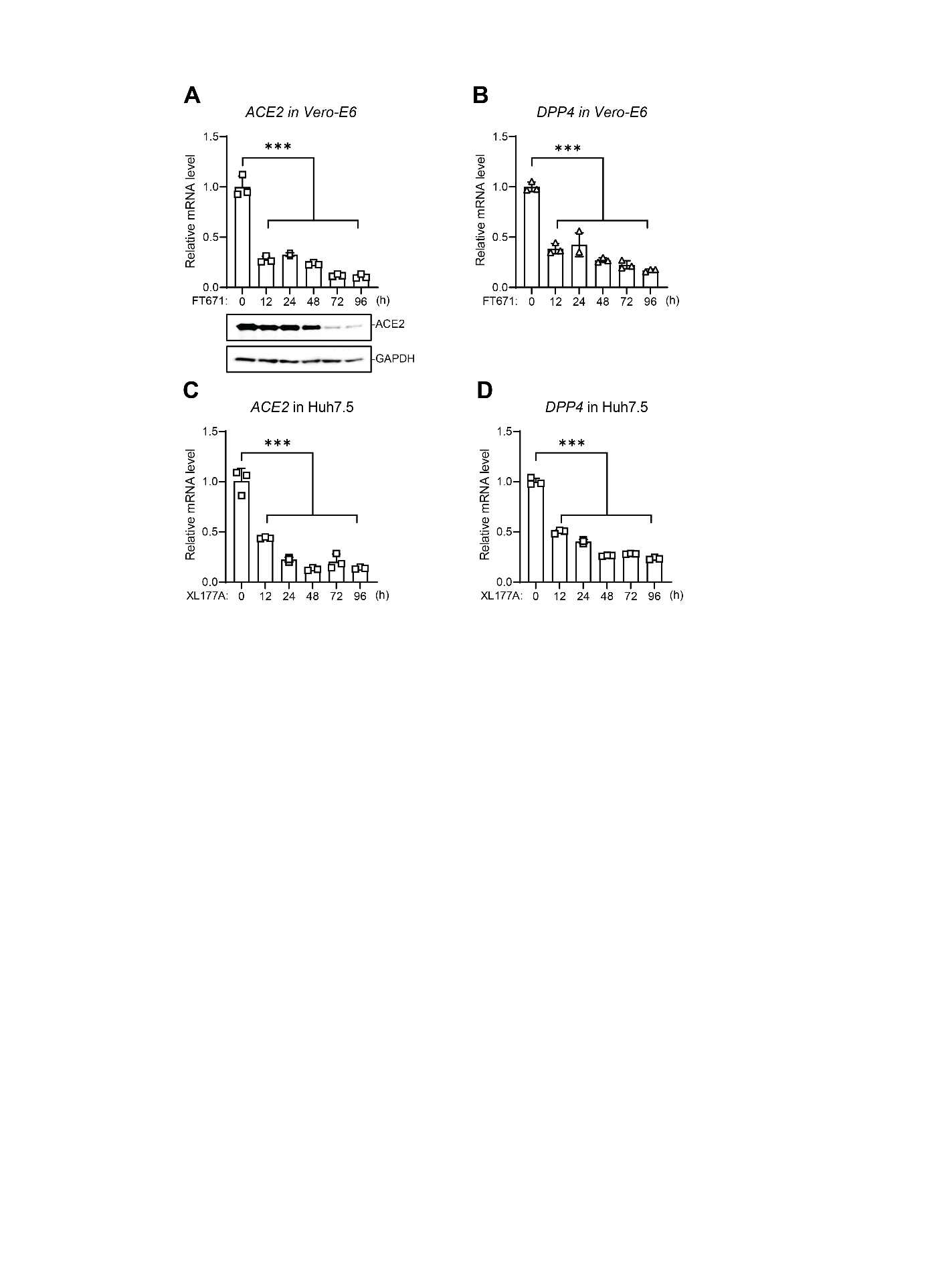


**Figure S5.** Inhibition of USP7 reduces viral receptor expression. (A-B) Inhibition of USP7 inhibits ACE2 and DPP4 expression. Vero E6 cells were treated with FT671 at 1.25 μM for the indicated times, ACE2 (A) and DPP4 (B) levels were measured by qPCR and western blot respectively. n=3. (C-D) Inhibition of USP7 inhibits ACE2 (C) and DPP4 (D) expression. Huh7.5 cells were treated with XL177A at 5 μM for the indicated times, ACE2 and DPP4 mRNA levels were measured by qPCR. n=3. The bar graphs show the mean ± SD of three independent experiments. n, number of independent experiments; One‐way ANOVA with Tukey’s test for (A to D); *** p < 0.001.

1. Tables

| **Table S1. The CRISPR sgRNA sequences** | |
| --- | --- |
| **LentiCRISPRv2 sgRNA** | **Target sequence** |
| USP7 sgRNA#1 | AGATGTATGATCCCAAAACG |
| USP7 sgRNA#2 | AGATTTCGCACAAAACACGG |
| RNF40 sgRNA#1 | GGGACGCCCAGCGATACAAG |
| RNF40 sgRNA#2 | CGCACTCGAAGCAGAAAACG |
| KCMF1 sgRNA#1 | GTACGTAGAATGTTTCACCC |
| KCMF1 sgRNA#2 | ACGCCGGGTTGCGTTGCGTG |
| ATG5 sgRNA | GGCCATCAATCGGAAACTCA |
| ATG7 sgRNA | CTTGTGTCCAAACCACCCTG |
| TAX1BP1 sgRNA#1 | CAACGTACGAGACAGAACGA |
| TAX1BP1 sgRNA#2 | GAGGAAACATTAACTCACAG |

| **Table S2. Oligos for qPCR in this study** | | |
| --- | --- | --- |
| **Oligos for qPCR** |  | **Sequence** |
| SARS-CoV-2 N1 | Forward | 5′-GACCCCAAAATCAGCGAAAT-3′ |
|  | Reverse | 5′-TCTGGTTACTGCCAGTTGAATCTG-3′ |
|  | Probe | 5′-6FAM-ACCCCGCATTACGTTTGGTGGACC-BHQ1-3′ |
| IAV-WSN PA | Forward | 5′-GGCCGACTACACTCTCGATGA-3′ |
|  | Reverse | 5′-TGTCTTATGGTGAATAGCCTGGTTT-3′ |
| ACE2 | Forward | 5′-GGGATCAGAGATCGGAAGAAGA-3′ |
|  | Reverse | 5′-AAGGAGGTCTGAACATCATCAGTG-3′ |
| DPP4 | Forward | 5′-GAATTATCCGGTCGAGTTTT-3′ |
|  | Reverse | 5′-GCCATCCTTTTAAAGAAGAG-3′ |
| KDM6A | Forward | 5′- TTCCTCGGAAGGTGCTATTCA-3′ |
|  | Reverse | 5′- GAGGCTGGTTGCAGGATTCA-3′ |
| ACTIN | Forward | 5′-GAGCACAGAGCCTCGCCTTT-3′ |
|  | Reverse | 5′-ATCATCATCCATGGTGAGCTGG-3′ |
| Mouse Actin | Forward | 5′- ACTGTCGAGTCGCGTCCA -3′ |
|  | Reverse | 5′- ATCCATGGCGAACTGGTGG-3′ |
| Mouse Ceacam1 | Forward | 5′-CCTCTATTCCAGGAAGTCTGGC-3′ |
|  | Reverse | 5′-GTTCAGGACAGTGTATGCGACG-3′ |
| ACE2 proximal enhancer | Forward | 5′-GCAACTGGAGGCTTAGTGAAAGA-3′ |
|  | Reverse | 5′-GTCCCTTGGAGGTCTGTGA-3′ |
| ACE2 distal enhancer | Forward | 5′-GCATGCCCCATTTCTGAACC-3′ |
|  | Reverse | 5′-TAAGGGGCATGGGACAGACT-3′ |
| ACE2 proximal promoter | Forward | 5′-TTCTAGACCTCTTTGGTCACTGTAAAATT-3′ |
|  | Reverse | 5′-ACCGGTACTTTTGGTTAATATTTTCC-3′ |
| ACE2 distal promoter | Forward | 5′-CGTCAGGTAGGCCCTTGAAC-3′ |
|  | Reverse | 5′-AATTTCAGAAGCGAGCTCAGTGT-3′ |

| **Table S3. Antibody information** | | |
| --- | --- | --- |
| **Antibody** | **Resource** | **Catalog** |
| ACE2 | Prosci | 3217 |
| KDM6A | Abclonal | A8159 |
| LC3 | Abclonal | A19665 |
| GAPDH | Abclonal | A19056 |
| Flag antibody | Abclonal | AE092 |
| HA antibody | Abclonal | AE065 |
| H3K27ac | Abcam | AB4729 |
| V5 antibody | Abclonal | AE089 |
| V5 antibody | Proteintech | 14440-1-AP |
| USP7 | Proteintech | 66514-1-Ig |
| β-Actin | Proteintech | 66009-1-Ig |
| TAX1BP1 | Proteintech | 14424-1-AP |
| RNF40 | Proteintech | 15621-1-AP |
| HRP-Goat Anti-Rabbit IgG(H+L) | Proteintech | SA00001-2 |
| HRP-Goat Anti-Mouse IgG(H+L) | Proteintech | SA00001-1 |

**Table S4. P values for each figure**

| **Figures** | **Sample** | **Comparison** | **p value** |
| --- | --- | --- | --- |
| Fig.1B | icSARS2-mNG | WT vs. USP7 KO#1 | <0.0001 |
|  |  | WT vs. USP7 KO#2 | <0.0001 |
| Fig.1C | SARS2 | 24hpi WT vs KO#1 | <0.0001 |
|  |  | 48hpi WT vs KO#2 | <0.0001 |
|  |  | 24hpi WT vs KO#1 | <0.0001 |
|  |  | 48hpi WT vs KO#2 | <0.0001 |
| Fig.1D | SARS2 | WT vs. USP7 KO#1 | <0.0001 |
|  |  | WT vs. USP7 KO#2 | <0.0001 |
|  | HKU5-SARS1-S | WT vs. USP7 KO#1 | <0.0001 |
|  |  | WT vs. USP7 KO#2 | <0.0001 |
|  | MERS | WT vs. USP7 KO#1 | <0.0001 |
|  |  | WT vs. USP7 KO#2 | <0.0001 |
| Fig.1E | SARS2-S | WT vs. USP7 KO#1 | <0.0001 |
|  |  | WT vs. USP7 KO#2 | <0.0001 |
|  | SARS1-S | WT vs. USP7 KO#1 | <0.0001 |
|  |  | WT vs. USP7 KO#2 | <0.0001 |
|  | MERS-S | WT vs. USP7 KO#1 | <0.0001 |
|  |  | WT vs. USP7 KO#2 | <0.0001 |
| Fig.1F | ACE2 | WT vs. KO | <0.0001 |
|  |  | WT vs. KO+WT | 0.2321 |
|  |  | KO vs. KO+WT | <0.0001 |
|  |  | KO+WT vs. KO+C223S | <0.0001 |
|  | DPP4 | WT vs. KO | <0.0001 |
|  |  | WT vs. KO+WT | 0.3245 |
|  |  | KO vs. KO+WT | <0.0001 |
|  |  | KO+WT vs. KO+C223S | <0.0001 |
| Fig.1G | SARS2 | WT vs. KO | <0.0001 |
|  |  | WT vs. KO+WT | 0.5429 |
|  |  | KO vs. KO+WT | <0.0001 |
|  |  | KO+WT vs. KO+C223S | <0.0001 |
|  | MERS | WT vs. KO | <0.0001 |
|  |  | WT vs. KO+WT | 0.3187 |
|  |  | KO vs. KO+WT | <0.0001 |
|  |  | KO+WT vs. KO+C223S | <0.0001 |
| Fig.1H | icSARS2-mNG | WT vs. KO | <0.0001 |
|  |  | WT vs. KO+WT | 0.0371 |
|  |  | KO vs. KO+WT | <0.0001 |
|  |  | KO+WT vs. KO+C223S | <0.0001 |
| Fig.1I | SARS-CoV-2 | WT vs. KO | 0.0002 |
|  |  | KO vs. KO+ACE2 | 0.0024 |
|  | VSV-SARS2-S | WT vs. KO | <0.0001 |
|  |  | KO vs. KO+ACE2 | 0.0008 |
| Fig.3A | SARS2=S | WT vs KDM6A KO | <0.0001 |
|  |  | WT vs DKO | <0.0001 |
| Fig.3C | KDM6A | WT vs. KO+EV | 0.9993 |
|  |  | KO+EV vs. KO+KDM6A | <0.0001 |
|  |  | KO+EV vs. KO+3KR | <0.0001 |
|  |  | KO+KDM6A vs. KO+3KR | 0.0003 |
|  | ACE2 | WT vs. KO+EV | <0.0001 |
|  |  | KO+EV vs. KO+KDM6A | 0.3234 |
|  |  | KO+EV vs. KO+3KR | <0.0001 |
|  |  | KO+KDM6A vs. KO+3KR | <0.0001 |
|  | DPP4 | WT vs. KO+EV | <0.0001 |
|  |  | KO+EV vs. KO+KDM6A | >0.9999 |
|  |  | KO+EV vs. KO+3KR | <0.0001 |
|  |  | KO+KDM6A vs. KO+3KR | 0.0095 |
| Fig.3D | rcVSV-SARS2-S | WT vs. KO+EV | <0.0001 |
|  |  | KO+EV vs. KO+KDM6A | 0.9518 |
|  |  | KO+EV vs. KO+3KR | 0.0002 |
|  |  | KO+KDM6A vs. KO+3KR | 0.0003 |
| Fig.3E | ACE2 enhancer | proximal | 0.0055 |
|  |  | Distal | 0.0001 |
|  | ACE2 promoter | proximal | <0.0001 |
|  |  | Distal | <0.0001 |
| Fig.3F | ACE2 enhancer | proximal | <0.0001 |
|  |  | Distal | <0.0001 |
|  | ACE2 promoter | proximal | 0.0819 |
|  |  | Distal | 0.0001 |
| Fig.4H | ACE2 | WT vs. KO+EV | <0.0001 |
|  |  | WT vs. KO+WT | 0.2 |
|  |  | KO+EV vs. KO+WT | <0.0001 |
|  |  | KO+WT vs. KO+RING | <0.0001 |
|  | DPP4 | WT vs. KO+EV | <0.0001 |
|  |  | WT vs. KO+WT | 0.0864 |
|  |  | KO+EV vs. KO+WT | <0.0001 |
|  |  | KO+WT vs. KO+RING | 0.0001 |
| Fig.4I | SARS2 | WT vs. KO+EV | <0.0001 |
|  |  | WT vs. KO+WT | 0.8108 |
|  |  | KO+EV vs. KO+WT | <0.0001 |
|  |  | KO+WT vs. KO+RING | <0.0001 |
|  | SARS1 | WT vs. KO+EV | <0.0001 |
|  |  | WT vs. KO+WT | 0.5609 |
|  |  | KO+EV vs. KO+WT | <0.0001 |
|  |  | KO+WT vs. KO+RING | <0.0001 |
|  | MERS | WT vs. KO+EV | <0.0001 |
|  |  | WT vs. KO+WT | 0.9849 |
|  |  | KO+EV vs. KO+WT | <0.0001 |
|  |  | KO+WT vs. KO+RING | <0.0001 |
| Fig.5J | KDM6A | Control vs. TAX1BP1-sg#1 | 0.6313 |
|  |  | Control vs. TAX1BP1-sg#2 | 0.0853 |
|  | ACE2 | Control vs. TAX1BP1-sg#1 | <0.0001 |
|  |  | Control vs. TAX1BP1-sg#2 | <0.0001 |
|  | DPP4 | Control vs. TAX1BP1-sg#1 | <0.0001 |
|  |  | Control vs. TAX1BP1-sg#2 | <0.0001 |
| Fig.5K | VSV-SARS1-S | Control vs. TAX1BP1-sg#1 | <0.0001 |
|  |  | Control vs. TAX1BP1-sg#2 | <0.0001 |
|  | VSV-SARS2-S | Control vs. TAX1BP1-sg#1 | <0.0001 |
|  |  | Control vs. TAX1BP1-sg#2 | <0.0001 |
|  | VSV-MERS-S | Control vs. TAX1BP1-sg#1 | <0.0001 |
|  |  | Control vs. TAX1BP1-sg#2 | <0.0001 |
| Fig.6A | ACE2 | 0 vs. 1.25 | <0.0001 |
|  |  | 0 vs. 2.5 | <0.0001 |
|  | DPP4 | 0 vs. 1.25 | <0.0001 |
|  |  | 0 vs. 2.5 | <0.0001 |
| Fig.6c | SARS2-S | 0 vs. 1.25 | <0.0001 |
|  |  | 0 vs. 2.5 | <0.0001 |
|  | MERS-S | 0 vs. 1.25 | <0.0001 |
|  |  | 0 vs. 2.5 | <0.0001 |
| Fig.6d | Huh7.5 | 0 vs. 1.25 | 0.0002 |
|  |  | 0 vs. 2.5 | <0.0001 |
|  | Calu-3 | 0 vs. 1.25 | 0.002 |
|  |  | 0 vs. 2.5 | <0.0001 |
| Fig.6E | ACE2 | 0 vs. 7.5 | <0.0001 |
|  |  | 0 vs. 15 | <0.0001 |
|  | DPP4 | 0 vs. 7.5 | <0.0001 |
|  |  | 0 vs. 15 | <0.0001 |
| Fig.6F | SARS2-S | 0 vs. 7.5 | <0.0001 |
|  |  | 0 vs. 15 | <0.0001 |
|  | MERS-S | 0 vs. 7.5 | <0.0001 |
|  |  | 0 vs. 15 | <0.0001 |
| Fig.6G | ACE2 | WT |  |
|  |  | DMSO vs. FT671 | <0.0001 |
|  |  | DMSO vs. XL177A | <0.0001 |
|  |  | KDM6A KO |  |
|  |  | DMSO vs. FT671 | 0.1464 |
|  |  | DMSO vs. XL177A | 0.5244 |
|  | DPP4 | WT |  |
|  |  | DMSO vs. FT671 | <0.0001 |
|  |  | DMSO vs. XL177A | <0.0001 |
|  |  | KDM6A KO |  |
|  |  | DMSO vs. FT671 | 0.1668 |
|  |  | DMSO vs. XL177A | 0.9716 |
| Fig.6H | SARS-CoV-2 | WT |  |
|  |  | DMSO vs. FT671 | <0.0001 |
|  |  | DMSO vs. XL177A | <0.0001 |
|  |  | KDM6A KO |  |
|  |  | DMSO vs. FT671 | 0.9351 |
|  |  | DMSO vs. XL177A | 0.8957 |
| Fig.6I | FT671 | 0 vs. 1.25 | 0.0017 |
|  |  | 0 vs. 2.5 | 0.0007 |
|  | XL177A | 0 vs. 7.5 | 0.0047 |
|  |  | 0 vs. 15 | 0.0008 |
| Fig.6J | FT671 | 0 vs. 1.25 | 0.0023 |
|  |  | 0 vs. 2.5 | <0.0001 |
|  | XL177A | 0 vs. 7.5 | 0.0005 |
|  |  | 0 vs. 15 | <0.0001 |
| Fig.6K | FT671 | 0 vs. 1.25 | <0.0001 |
|  |  | 0 vs. 2.5 | <0.0001 |
| Fig.7A | ACE2 | DMSO vs. FT671 2.5μM | <0.0001 |
|  |  | DMSO vs. FT671 5μM | <0.0001 |
|  | DPP4 | DMSO vs. FT671 2.5μM | <0.0001 |
|  |  | DMSO vs. FT671 5μM | <0.0001 |
| Fig.7B | viral RNA | DMSO vs. FT671 | <0.0001 |
|  |  | DMSO vs. Remdesivir | <0.0001 |
|  | plaque assay | DMSO vs. FT671 | <0.0001 |
|  |  | DMSO vs. Remdesivir | <0.0001 |
| Fig.7C | viral RNA | DMSO vs. FT671 | <0.0001 |
|  |  | DMSO vs. Remdesivir | <0.0001 |
|  | plaque assay | DMSO vs. FT671 | <0.0001 |
|  |  | DMSO vs. Remdesivir | <0.0001 |
| Fig.7D | IAV | DMSO vs. FT671 | 0.5 |
| Fig.7E | WA1 | DMSO vs. FT671 | <0.0001 |
|  |  | DMSO vs. Remdesivir | <0.0001 |
|  | E802D | DMSO vs. FT671 | <0.0001 |
|  |  | DMSO vs. Remdesivir | 0.0034 |
|  |  | FT671vs. Remdesivir | 0.0023 |
| Fig.7F | WA1 | DMSO vs. FT671 | <0.0001 |
|  | alpha | DMSO vs. FT671 | <0.0001 |
|  | beta | DMSO vs. FT671 | <0.0001 |
|  | gamma | DMSO vs. FT671 | <0.0001 |
|  | delta | DMSO vs. FT671 | <0.0001 |
|  | omicron | DMSO vs. FT671 | <0.0001 |
| Fig.7G | ACE2 | DMSO vs. FT671 | <0.0001 |
|  | DPP4 | DMSO vs. FT671 | 0.00017 |
| Fig.7H | SARS2 | DMSO vs. FT671 | 0.00038 |
|  | MERS | DMSO vs. FT671 | 0.00037 |
| Fig.8D | SI | DMSO vs. FT671 | <0.0001 |
|  | Spleen | DMSO vs. FT671 | <0.0001 |
|  | Liver | DMSO vs. FT671 | <0.0001 |
|  | Lung | DMSO vs. FT671 | <0.0001 |
| Fig.8E | Spleen | DMSO vs. FT671 | <0.0001 |
| Fig.8F | Liver | DMSO vs. FT671 | <0.0001 |
| Fig.8G | Lung | DMSO vs. FT671 | <0.0001 |
| Fig.8H | Spleen | DMSO vs. FT671 | 0.0005 |
| Fig.8I | Liver | DMSO vs. FT671 | 0.0006 |
| Fig.8J | Lung | DMSO vs. FT671 | 0.0035 |
